# Unveiling New Triazoloquinoxaline‐Based PROTACs Designed for the Selective Degradation of the ncBAF Chromatin Remodeling Subunit BRD9

**DOI:** 10.1002/chem.202404218

**Published:** 2025-05-20

**Authors:** Martina Pierri, Guglielmo Bove, Erica Gazzillo, Ester Colarusso, Francesca Scala, Giacomo Pepe, Stefania Terracciano, Maria Giovanna Chini, Nicla Simonelli, Ines Bruno, Angela Nebbioso, Pietro Campiglia, Giuseppe Bifulco, Lucia Altucci, Nunzio Del Gaudio, Gianluigi Lauro

**Affiliations:** ^1^ Department of Pharmacy University of Salerno Via Giovanni Paolo II 132 Fisciano 84084 Italy; ^2^ Department of Precision Medicine University of Campania “Luigi Vanvitelli” Vico L. De Crecchio 7 Naples 80138 Italy; ^3^ PhD Program in Drug Discovery and Development University of Salerno Via Giovanni Paolo II 132 Fisciano 84084 Italy; ^4^ Department of Biosciences and Territory University of Molise C.da Fonte Lappone Pesche 86090 Italy; ^5^ Program of Medical Epigenetics Vanvitelli Hospital Naples Italy; ^6^ BIOGEM Via Camporeale Area P.I.P. Ariano Irpino (AV) Italy; ^7^ Department of Life Sciences Health and Health Professions Link Campus University Via del Casale di San Pio V, 44 Rome 00165 Italy

**Keywords:** BRD9, cancer, drug design, PROTAC, triazoloquinoxaline compounds

## Abstract

PROteolysis Targeting Chimera (PROTAC) technology is an innovative and potent approach for achieving targeted protein degradation (TPD). Within bromodomain‐containing proteins, various degraders targeting BET family‐related targets, for example, BRD4, were developed in the last years. On the other hand, a limited number of PROTACs acting against non‐BET proteins were reported so far. Among them, BRD9 was recently linked to oncogenic roles in the tumorigenesis processes, especially in sarcomas and leukemias. Herein, we describe the design and synthesis of a focused collection of new BRD9‐targeting degraders based on the [1,2,4]triazolo[4,3‐*a*]quinoxaline heterocyclic scaffold employing two distinct E3 ubiquitin ligase ligands. Through in silico evaluation, synthesis, binding affinity determination, and in vitro analysis, we identified two new VHL‐based PROTACs (**2** and **9**), which showed remarkable degradation of the protein of interest and antiproliferative activity in acute myeloid leukemia (AML) cells. Notably, compound **9** exhibited selective degradation of BRD9 over BRD7. These results enlarge and differentiate the pool of heterobifunctional molecules able to degrade BRD9 through the proteasome machinery, providing a promising reference for the discovery of new tools to further explore both the involvement of this epigenetic regulatory factor in tumor processes and to evaluate novel strategies for AML treatment.

## Graphical abstract

We present new BRD9‐targeting degraders featuring a chemical warhead and E3 ligase ligand that distinctly differ from the typical chemical units used for BRD9 degradation. Specifically, we identified two VHL‐based PROTACs (**2** and **9**) based on a [1,2,4]triazolo[4,3‐*a*]quinoxaline scaffold that effectively and selectively degrade BRD9 and exhibit antiproliferative effects in acute myeloid leukemia (AML) cells, offering potential strategies for AML treatment and insights into BRD9's role in tumors.

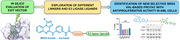

## 1 Introduction

In the last decades, the scientific community has devoted even higher attention to the development of novel therapeutic strategies to bypass limitations of small molecules for cancer treatment and to reach a high target occupancy and high selectivity toward the targets of interest. In this field, considering that many proteins involved in tumorigenesis comprise multiple domains or are part of complexes comprising different macromolecules, the use of small molecules targeting specific subunits does not often lead to phenotypic efficacy.^[^ ^1^, ^2^ ^]^

Among the newly explored techniques in cancer therapy, the PROteolysis Targeting Chimeras (PROTAC) strategy is certainly one of the most exciting and innovative.^[^ ^3^, ^4^ ^]^ In recent years, PROTAC technology has been identified as a novel drug discovery strategy able to offer therapeutic interventions otherwise not achievable through conventional medicinal chemistry approaches, showing advantageous results especially for proteins previously described as undruggable.^[^ ^5^, ^6^, ^7^, ^8^ ^]^ This innovative technology employs small molecules able to degrade the target protein rather than modulate its function. Specifically, a ligand (also called a “warhead”) targeting a protein of interest (POI) is connected to an E3 ubiquitin ligase linker by a linker of different lengths, rigidity/flexibility, and chemical nature.

**Figure 1:**
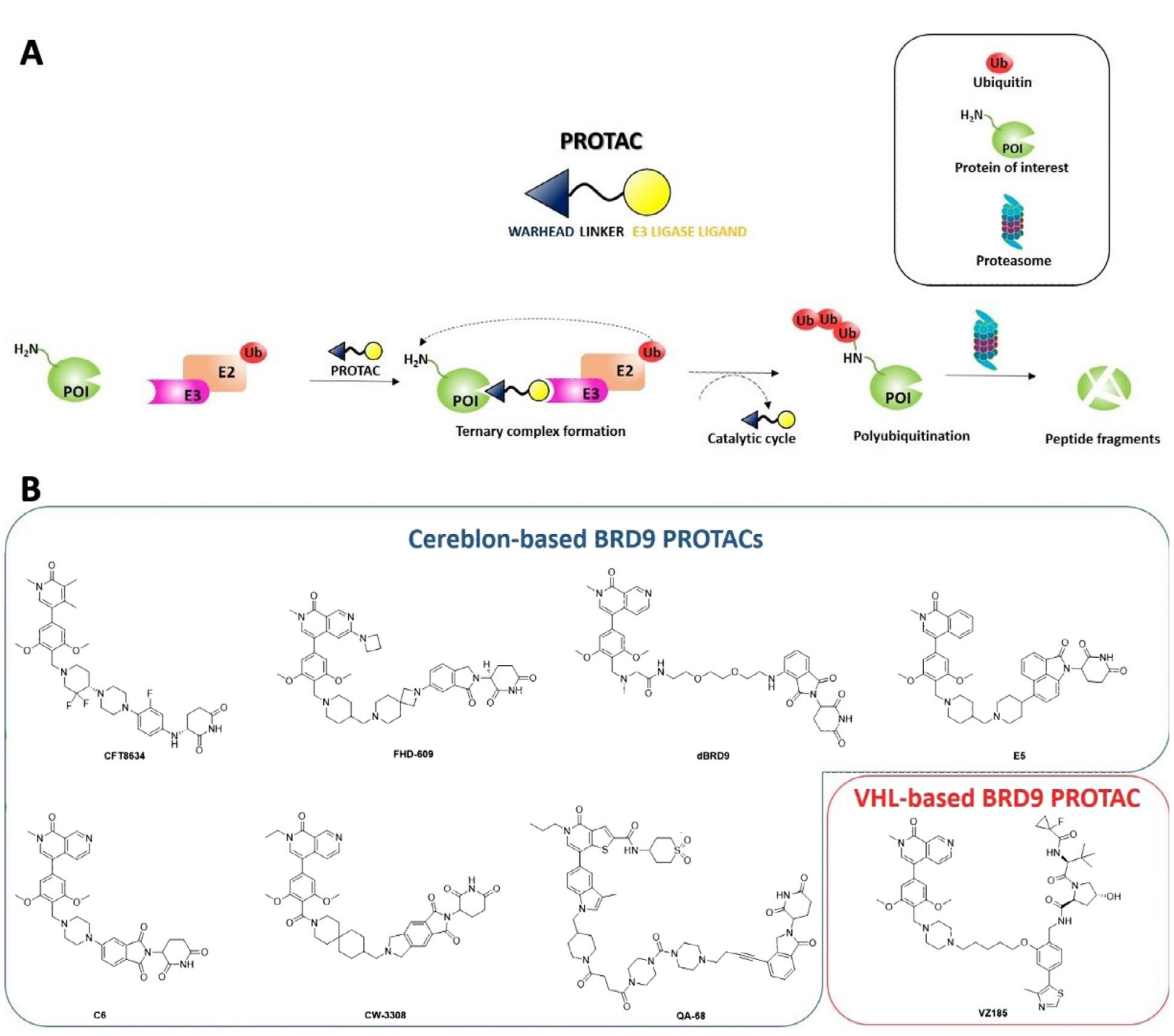
(A) Schematic depiction of the mechanism of action of PROTACs. (B) Chemical structures of cereblon‐ and VHL‐based BRD9 PROTACs so far disclosed.

In this way, the target protein is brought close to the E3 ligase that promotes its polyubiquitination and degradation by proteasome activity through a catalytic and sub‐stoichiometric mechanism of action (Figure 1A). However, the modular nature of PROTACs requires a proper exploration of the chemical space involving the choice of ligands for the target protein, of the E3 ligase ligands, and of the linkers. All these variables tightly control structure‐activity relationships and physicochemical properties of PROTACs, impacting their degradation activity in vitro and in vivo.^[^ ^9^, ^10^, ^11^, ^12^, ^13^, ^14^ ^]^ However, although the PROTAC strategy has shown great potential and numerous advantages, the design of new degraders is predominantly an iterative process, with structure optimization guided mostly by trial‐and‐error approaches because of the limited availability of ternary complex crystal structures.^[^ ^15^ ^]^ Thus, the requirement to identify more systematic methods, for example, based on in silico techniques, is of critical importance to drive PROTAC design.^[^ ^16^, ^17^, ^18^, ^19^ ^]^

To confirm the success of the targeted protein degradation and underline the power of this approach in tumors, Arvinas Therapeutics started the first‐ever clinical study of a PROTAC (ARV‐110) in 2019. This compound is an orally bioavailable degrader of the androgen receptor (AR), developed to treat metastatic castration‐resistant prostate cancer.^[^ ^20^ ^]^ Additionally, there has been significant interest from the scientific community in the development of PROTACs that target epigenetic proteins, particularly bromodomain and extra‐terminal (BET) proteins (BET), for example, bromodomain‐containing protein 4 (BRD4),^[^ ^21^ ^]^ and non‐BET proteins, for example, bromodomain‐containing protein 9 (BRD9). In recent years, the discovery of highly potent and selective pan‐BET and BRD4‐targeting PROTACs has clearly demonstrated that BET protein inhibition and degradation result in significantly different biological effects. These advances underscore the potential of PROTAC degraders as an innovative therapeutic avenue for targeting BRD family proteins.^[^ ^22^ ^]^

To date, a set of PROTACs, mostly based on the Cereblon E3 ligase recruiting moiety, have been developed for the degradation of BRD9 (Figure 1B), featuring promising outcomes in cancer therapies,^[^ ^23^, ^24^, ^25^, ^26^, ^27^, ^28^, ^29^, ^30^, ^31^, ^32^ ^]^ and currently two degraders have entered clinical trials, namely **FHD‐609** (NCT04965753) and **CFT8634** (NCT05355753).^[^ ^33^ ^]^ Unfortunately, the two clinical trials with BRD9 PROTACs were terminated, thus highlighting the urgent need of new BRD9 PROTACs. The targeted protein degradation of BRD9 is very attractive since the classical protein binding and the inhibition of its function in reading histone coding, in contrast to genetic knockdown, have not always resulted in a significant phenotypic response. The main limitation of BRD inhibitors is that these epigenetic proteins typically contain multi‐domain arrangements and are frequently components of multi‐subunit complexes. Hence, traditional bromodomain inhibitors do not fully ablate oncogenic transcription.^[^ ^34^, ^35^ ^]^

In this scenario, we pursued and enlarged the chemical variability of this innovative technology, developing novel PROTAC degraders based on our recently discovered triazoloquinoxaline binders employed as new and efficient warheads to recruit BRD9 protein.^[^ ^36^, ^37^ ^]^ In more detail, ligands based on this core were identified thanks to the application of in silico tools developed by us,^[^ ^36^, ^37^, ^38^, ^39^, ^40^, ^41^ ^]^ and they emerged as valid starting points for the development of new BRD9 PROTACs. Specifically, to develop new promising degraders, BRD9 3D structure‐based pharmacophore models coupled with molecular docking were here used to detect the suitable attachment position of the linker without impairing the respect of pharmacophoric features necessary for the binding to the protein of interest. Here we propose a successful workflow based on in silico, synthetic, and in vitro approaches for the development of BRD9‐degrading PROTACs.

## 2 Results and Discussion

**Figure 2:**
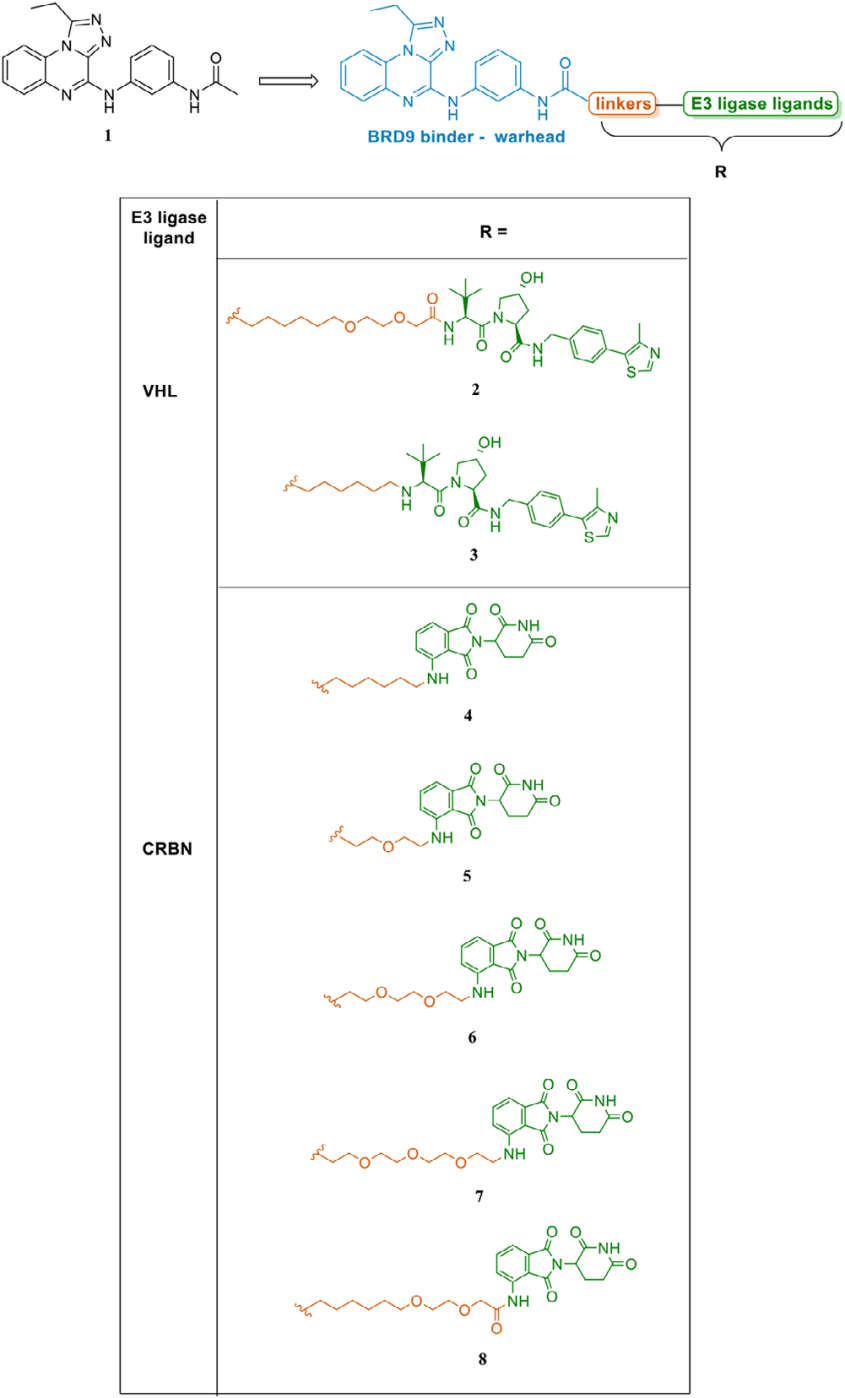
Chemical structures of VHL‐ and CRBN‐based BRD9 PROTACs (**2**−**8**) explored in this study, presenting the potent binder **1** employed as a warhead.

**Figure 3:**
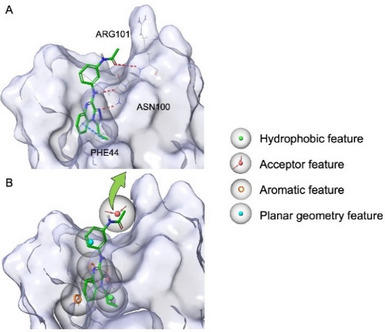
(A) Binding mode of compound **1** in the BRD9 binding pocket predicted through molecular docking experiments and fundamental interaction with key residues. H bonds and π─π interactions are reported in red and blue dotted lines, respectively. (B) Compound **1** superimposed with the “pharm‐druglike 2″ BRD9 pharmacophore model employed. The solvent‐exposed *N*‐phenylacetamide moiety was used as an exit vector (green arrow) for linker conjugation.

**Scheme 1:**
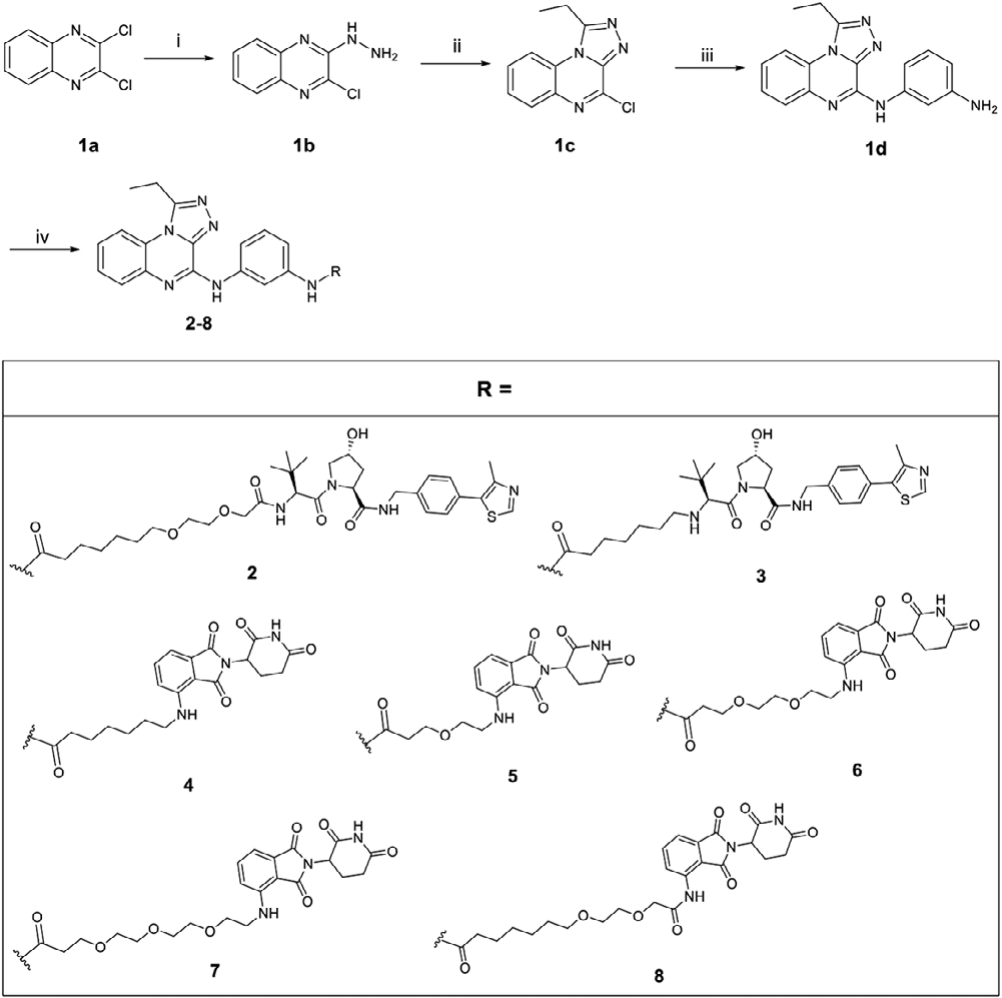
Syntheses of compounds **2**−**8**. Reagents and conditions: (i) hydrazine monohydrate, EtOH, room temperature, overnight, (ii) CH_3_CH_2_C(OC_2_H_5_)_3_, room temperature, overnight, (iii) *m*‐phenylenediamine, 110 °C MW, 8 minutes, DMSO, and (iv) RCOOH, HATU, TEA, DMF, room temperature, 1 hour.

Compound **1** (Figure 2), previously disclosed by us, was chosen as a warhead based on its promising affinity for BRD9 (IC_50_ = 4.20 ± 1.92 µM) and, above all, selectivity over a panel of other bromodomain proteins.^[^ ^36^ ^]^ Starting from this compound, we initiated a PROTAC campaign designing a small set of derivatives (compounds **2**−**8**), shown in Figure 2. The binding mode of **1** into the BRD9 pocket, obtained through molecular docking and pharmacophore screening with our BRD9 3D structure‐based pharmacophore model (“pharm‐druglike 2”, Figure 3),^[^ ^36^ ^]^ highlighted the solvent‐exposed *N*‐phenylacetamide moiety, which, for this reason, was selected as an exit vector to conjugate an appropriate linker readily. Specifically, since this moiety was included in the acceptor feature of the “pharm‐druglike 2” model and its functionalization does not affect the respect of the pharmacophoric points, we decided that linkers for PROTAC building were to be added via amide bond formation without impairing the binding to the target protein. Despite the crucial role of linkers in the overall degradation activity, there is currently no generally applicable rule for de novo PROTAC linker design.^[^ ^15^, ^42^ ^]^ Hence, often structurally simple alkyl or PEG chains are often used as starting points, considering the importance of a certain degree of flexibility to achieve the correct folding to drive the ternary complex formation.^[^ ^15^, ^43^ ^]^ As shown in Figure 2, linkers (orange) with both different lengths and PEG units were selected to explore a broad range of chemical space and properties. In more detail, as shown in Figure 2, we employed hydrophobic alkyl linkers (see compounds **3** and **4**), and linkers featuring one to three PEG units (see compounds **2** and **5**–**8**), considering that the presence of oxygen atoms could be beneficial to degradation activity.^[^ ^23^ ^]^ Additionally, two well‐established ligands for E3 ubiquitin ligases (shown in green) were used to generate this initial set of PROTACs, targeting both the von Hippel−Lindau (VHL) and cereblon (CRBN) E3‐ligases, that are VH032^[^ ^44^ ^]^ and thalidomide, respectively.^[^ ^45^ ^]^ Compounds **2−8** were then synthesized via a convenient synthetic procedure, shown in Scheme 1. The key intermediate triazoloquinoxaline core **1d** was obtained in high yields as already described in our recent work.^[^ ^36^ ^]^ Compound **1d** was then conjugated to the primary amine functionality using commercially available building blocks, consisting of a linker and E3 ligase ligands, by amide bond formation with HATU as the coupling reagent, resulting in the final compounds **28**. Detailed synthetic procedures are described in the Experimental Section and Supporting Information, and each compound was fully characterized by NMR and high‐resolution mass spectrometry (HRMS) experiments.

To evaluate the suitability of the vector point for linker conjugation, the binding of compound **2**, chosen as a pilot compound of the series, was assessed against BRD9 through AlphaScreen assay, confirming the interaction with the POI. In more detail, compound **2** led to 21.6 ± 0.3 % residual binding of histone H4 at 10 µM ligand concentration, reflected with an IC_50_ value of 2.97 ± 0.25 µM (Figure S25, Supporting Information), thus indicating that both the POI warhead and the related chosen attachment point for the linkers and ligase ligands were compatible with preserving binding toward BRD9 and with the development of PROTACs.

**Figure 4:**
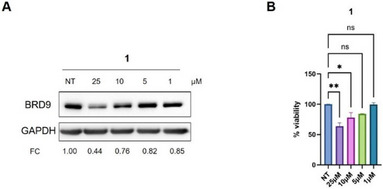
(A) WB analysis indicating BRD9 protein levels in U937 cells following 48‐hours treatments with the BRD9 inhibitor **1** at the indicated concentrations with relative quantification. (FC: fold change over GAPDH). (B) Phenotypic effect of the BRD9 inhibitor **1** in U937 cells. The CCK8 viability assay was performed following treatments with the indicated compound at 48 hours in U937 cells (*P < 0.05, **P < 0.01).

**Figure 5:**
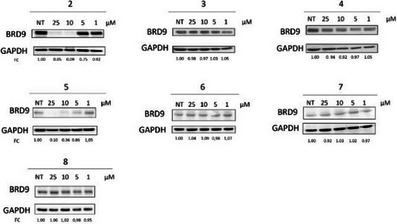
Degradation effects of BRD9 PROTACs in U937 cells. WB analysis showing BRD9 protein levels following 48‐hours treatments with compounds **2**−**8** at the indicated concentrations with relative quantification. (FC: fold change over GAPDH).

Based on these encouraging results, we further investigated the biological profile of all synthesized PROTACs **2**−**8** by western blotting (WB) to assess their degradation activity in a pro‐monocytic human myeloid leukemia cell line (U937), which is sensitive to BRD9 depletion.^[^ ^1^ ^]^ In addition, to compare the PROTACs‐induced degradation with that of classical inhibitors, we evaluated BRD9 protein levels after treatment with compound **1**, which served as the warhead for the designed degraders. Specifically, cells were treated with **1** (Figure 4A) and PROTACs **2−8** (Figure 5) for 48 hours at different concentrations. Consistent with previously reported findings that assessed the ability of BRD9 to regulate its own expression and the poor phenotypic response shown following BRD9 inhibition,^[^ ^1^, ^35^, ^46^ ^]^ treatment with the inhibitor **1** resulted in only a slight reduction in BRD9 protein levels (Figure 4A). Additionally, U937 cell viability was minimally affected, showing a moderate cytotoxic effect only at high concentrations of **1** (Figure 4B).

The use of PROTACs revealed substantial differences in outcomes due to the advantages of targeted protein degradation. Importantly, results showed that compounds **2** and **5** strongly reduced BRD9 protein levels in a dose‐dependent manner; conversely, no effect was observed following treatment with the remaining compounds **3**, **4**, **6**, **7**, and **8** (Figure 5). Importantly, the comparison between the two VHL‐based compounds **2** and **3** reveals that the incorporation of PEG units in compound **2** results in a markedly different degradation profile compared to compound **3**, which features an alkyl hydrophobic chain as a linker. On the other hand, the introduction of various PEG chains in CRBN‐based compounds **6–8** did not result in any degradation of the target protein, with the only exception of compound **5**, which showed a moderate reduction in protein levels. Interestingly, compounds **2** and **8**, which share the same linker but differ in their E3 ligase‐recruiting moieties, exhibited markedly different BRD9 degradation profiles. This highlights that the overall degradation efficiency depends on the careful combination of the warhead and E3 ligase ligand, properly connected by an appropriate linker.

**Figure 6:**
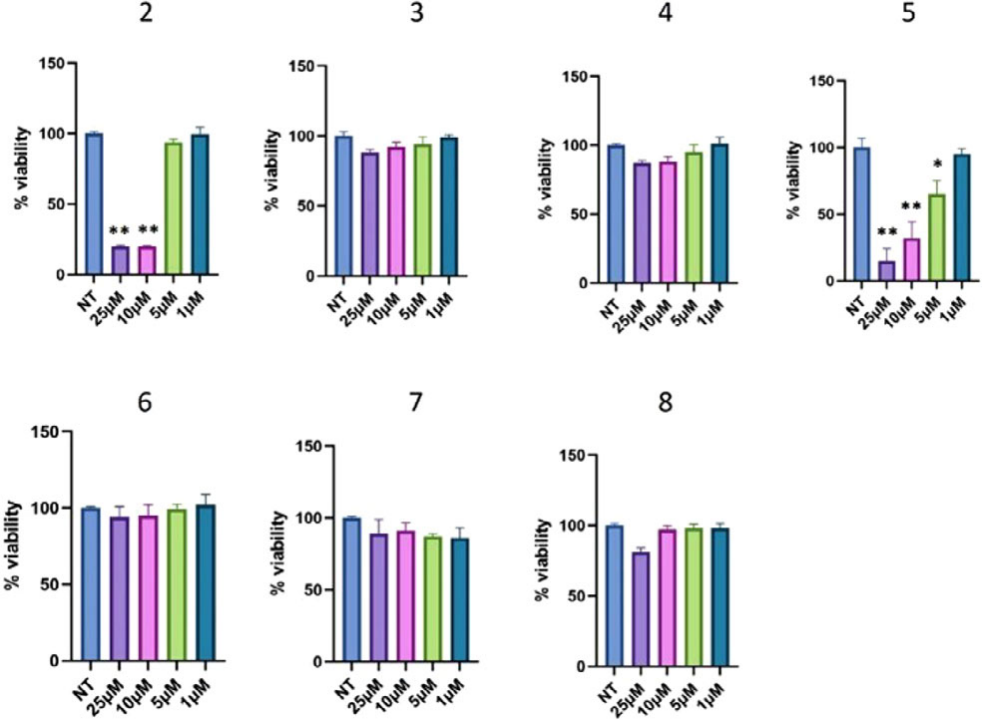
Phenotypic effects of BRD9 PROTACs in U937 cells. The CCK8 viability assay was performed following treatments with the indicated compounds at 48 hours in U937 cells (*P < 0.05, **P < 0.01).

Notably, the degradation activity of the two active PROTACs **2** and **5** on treated U937 cells corresponded to a strong reduction of cell viability (Figure 6), highlighting the remarkable efficiency of cell growth suppression achieved by BRD9 degradation compared to bromodomain inhibition. As expected, we did not observe variation in cell viability following treatment with the inactive compounds **3**, **4**, **6**, **7**, and **8** (Figure 6).

The encouraging results led us to further optimize the degradation profile of the best degrader of the series, that is, compound **2** featuring the PEGylated linker and the VHL‐recruiting moiety. This choice was also driven by the fact that compound **5**, being a CRBN‐based PROTAC, could display off‐target effects in AML and other hematological malignancies due to the neomorphic activity of CRBN ligands resulting in the degradation of IKZF1/3 and GSPT1 proteins.^[^ ^47^, ^48^, ^49^, ^50^ ^]^

**Scheme 2:**
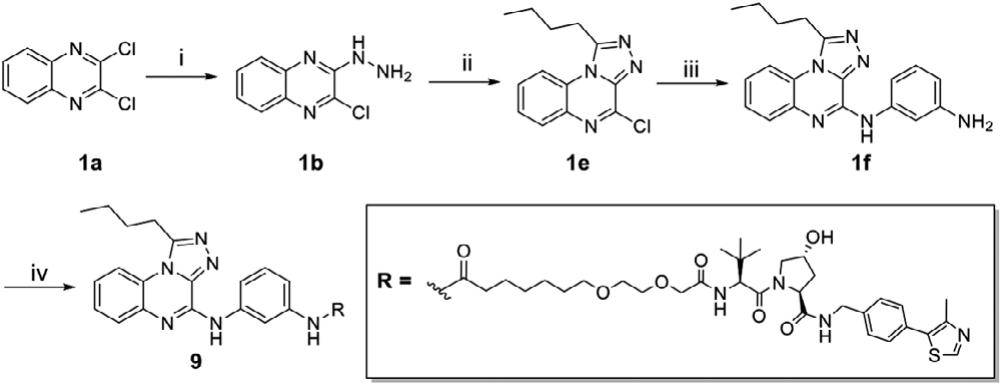
Synthesis of compound **9**. Reagents and conditions: (i) hydrazine monohydrate, EtOH, room temperature, overnight, (ii) 1,1,1‐triethoxypentane, room temperature, overnight, (iii) *m*‐phenylenediamine, 110 °C MW, 6 minutes, DMSO, and (iv) RCOOH, HATU, TEA, DMF, room temperature, 1 hour.

In parallel with this project on new degraders of BRD9, we recently discovered that, exploiting BRD9 structural features, the introduction of a longer alkyl chain on the C‐1 position of the triazoloquinoxaline core (representing the acetyl lysine mimetic moiety) leads to an enhancement of both the activity and the selectivity of BRD9 over other bromodomains, comprising the highly homologous bromodomain‐containing protein 7 (BRD7).^[^ ^37^, ^51^ ^]^ In particular, this evidence is based on the fact that BRD9 is able to accommodate bulkier substituents in its binding pocket than the other bromodomains, due to the presence of a conserved network of water molecules that can be perturbed by hydrophobic groups.^[^ ^52^ ^]^ This led us to design a new PROTAC (compound **9**, Scheme 2) featuring the butyl substituent at C‐1, inspired by our previously identified BRD9 selective hit compound.^[^ ^37^ ^]^ For this reason, compound **9** was synthesized in high yields following the similar synthetic route previously described, as shown in Scheme 2.

Accordingly, as with the previous compound **2**, we confirmed the BRD9 binding for **9** through AlphaScreen assay. Interestingly, we detected a 4.5‐fold higher binding affinity to the protein‐of‐interest compared to the initial degrader **2** with an IC_50_ value of 0.66 ± 0.04 µM after treatment with compound **9** (Figure S25, Supporting Information).

**Figure 7:**
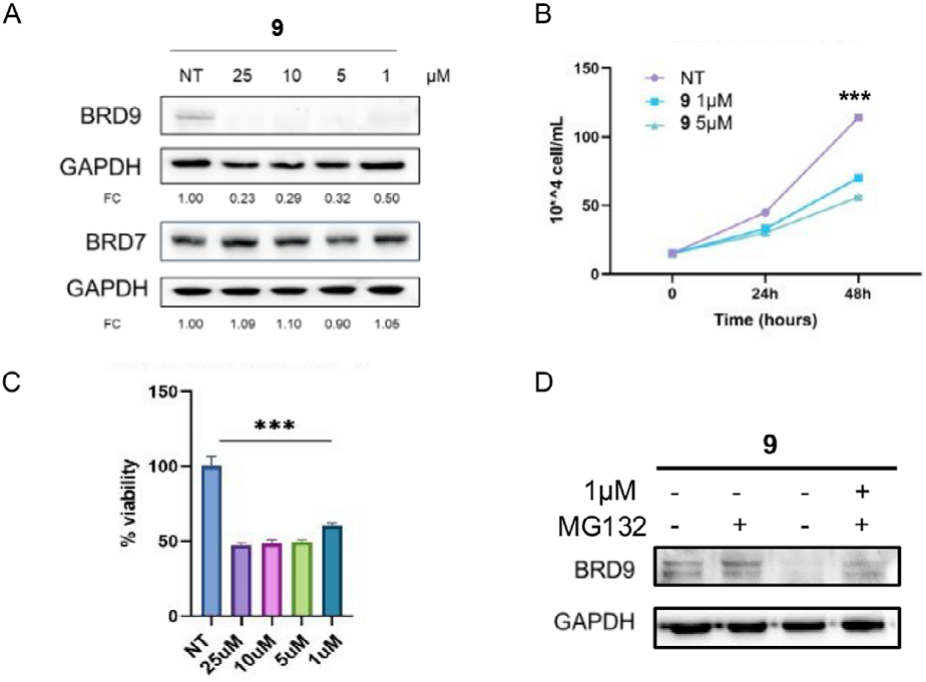
Compound **9** selectively degrades BRD9 inducing cell proliferation arrest. (A) WB analysis showing BRD9 and BRD7 protein levels following 48‐hours treatment with compound **9** at indicated concentrations with relative quantification. (FC: fold change over GAPDH) in U937 cells. B) Proliferation assay of U937 cells following time‐point treatment with compound **9** (***P < 0.001). C) CCK8 viability assay performed in U937 cells following 48‐hours treatments with the compound **9** (***P < 0.001). D) WB analysis of BRD9 protein levels observed following 48‐hours treatment with compound **9** at indicated concentration with or without MG132 (25 µM for 6 hours) in the U937 cell line.

The obtained PROTAC **9** featuring the butyl substituent on the C‐1 position was then biologically evaluated by western blotting. In line with the increased affinity detected, compound **9** effectively induced BRD9 degradation at a concentration as low as 1 µM, revealing a higher potency compared to compound **2** (Figure 7A). As expected, this trend correlates with the higher BRD9 recruitment measured for compound **9**. Remarkably, **9** was able to selectively degrade BRD9 while not interfering with BRD7 protein levels (Figure 7A), thus corroborating the importance of employing bulky substituents (for example, a butyl group) in the acetyl lysine mimetic moiety in gaining affinity and selectivity toward BRD9. To further explore its anticancer potential, compound **9** was evaluated for its antiproliferative activity on the U937 cancer cell line. Treatment with **9** significantly impaired leukemic cell viability and negatively affected AML cell proliferation (Figure 7B,C). To examine mechanisms of protein degradation with the best degraders, U937 cells were treated with proteasome inhibitor MG132 alongside PROTAC **9**.

As shown in Figure 7D, MG132 treatment rescued BRD9 depletion, indicating that the degradation occurred through a proteasome‐dependent mechanism. In addition, the well‐known BRD9 PROTAC **dBRD9** was investigated in vitro as a reference compound in the U937 cell line (Figure S26). Interestingly, the results outlined that, despite **dBRD9** effectively degraded BRD9, our lead degrader **9** demonstrated a superior ability to reduce leukemic cell viability and AML cell proliferation.

In order to further characterize the promising degraders **2** and **9**, a careful and extensive investigation of their pharmacokinetic properties was performed. First, we assessed the metabolic stability of compounds **2** and **9** following incubation with human plasma for up to 120 minutes. The results demonstrated a highly satisfactory level of stability, with at least 90% compound recovery for both test compounds (Table S1).

Furthermore, we investigated the microsomal stability of compounds **2** and **9** through incubation with human liver microsomes (HLMs). HLMs are subcellular fractions isolated from the liver's endoplasmic reticulum via differential high‐speed centrifugation. Hepatic microsomes contain a variety of enzymes, including cytochrome P450 enzymes, flavin monooxygenases, and certain phase II enzymes, such as specific isoforms of uridine 5′‐diphospho‐glucuronosyltransferase and epoxide hydrolase. Hepatic microsomes remain the simplest and most popular subcellular fractions used to perform metabolic stability studies.

In our assay we followed the loss of the test compounds over time under CYP‐mediated metabolic pathways. The extent of hepatic metabolism allowed us the determination of different pharmacokinetic parameters such as in vitro t1/2, CL_int, in vitro_ and CL_int, in vivo_. In vitro t1/2 and CL_int, in vitro_ of test compounds after liver microsomes incubation were calculated according to “well‐stirred” model. In the “well‐stirred” model, the liver is represented by a single compartment, where the unbound concentration of a drug leaving the liver is in equilibrium with the intracellular unbound concentration in the hepatocytes.^[^ ^53^ ^]^ Predicted CL_int, invivo_ values were determined using human PBSF.^[^ ^54^ ^]^

Our results indicated that compounds **2** and **9** share a similar metabolic stability (Table S1). According to the classification by McNaney et al.,^[^ ^55^ ^]^ our results showed that compounds **2** and **9** exhibited very high clearance (CL_int_ *_,_*_in vitro_ > 45 mL min^−1^ kg^−1^).

As the substrate underwent time‐dependent disappearance, various metabolites emerged. To further investigate these metabolic products, we analyzed the metabolites generated after incubation with HLMs. This was accomplished by employing a strategy that integrates high‐resolution MS/MS data with advanced processing algorithms in the Compound Discoverer software. The metabolites were tentatively characterized based on their accurate mass, fragmentation patterns, and retention times. Consistent with the similar microsomal stability of compounds **2** and **9**, LC‐MS/MS analysis revealed that their primary metabolites are generated through *O*‐dealkylation reactions (Figures S27–S29). This metabolic pathway involves the removal of an alkyl group from an oxygen atom, a process commonly catalyzed by cytochrome P450 enzymes in the liver.

Specifically, the **M1** metabolites of compounds **2** and **9** exhibited precursor ions [M─H]⁺ at *m/z* 433 (C_24_H_28_N_6_O_2_) and *m/z* 461 (C_26_H_32_N_6_O_2_), respectively (Figures S28A and S30A). Similarly, the **M3** metabolites showed ions at *m/z* 477 (C_26_H_32_N_6_O_3_) for compound **2** and *m/z* 505 (C_28_H_36_N_6_O_3_) for compound **9**, suggesting hydrolysis of the ether linker in both compounds. (Figures S28C–S30C).

The identification of the same **M2** and **M4** metabolites for compounds **2** and **9**, with precursor ions [M─H]⁺ at *m/z* 533 (C_26_H_36_N_4_O_6_S) and *m/z* 489 (C_24_H_32_N_4_O_5_S), respectively, confirms that the ether bond used to connect the linker to the two ligands is susceptible to metabolic reactions (Figures S28B, S28D, S30B, and S30D). This finding aligns with data reported in the literature, where ether bonds are known to undergo hydrolysis under certain conditions.^[^ ^56^ ^]^

Taken together, the findings here reported highlight the potency and selectivity of compound **9** in degrading BRD9 and arresting leukemic cell growth while not perturbing BRD7 levels, which represents a significant achievement in the field of the selective BRD9 targeting for cancer treatment. Not the least, compound **9** reported promising results considering its metabolic stability, making it a worthy candidate for future preclinical and clinical studies.

## 3 Conclusion

In the last few years, the identification of new BRD9 PROTACs emerged as an alternative pharmacological modality to address a more effective cellular activity than monovalent inhibitors. Findings reported in this work, obtained through a multidisciplinary approach based on computer‐aided and synthetic methodologies, highlighted the identification of a new class of potent VHL‐based degraders (**2** and **9**), which exhibited efficient protein degradation via the ubiquitin‐proteasome system. Importantly, these developed PROTACs feature warheads that consistently differ from the common chemical unit employed for BRD9 degradation (i.e., **dBRD9**, **C6**, and **VZ185**; see Figure 1B), thus representing a promising starting point for developing new generations of BRD9 PROTACs. Importantly, PROTACs‐induced BRD9 degradation promotes an antiproliferative phenotype in a pro‐monocytic human myeloid leukemia cell line (U937) better than the well‐known BRD9 degrader **dBRD9**, reinforcing the valid potential of this pharmaceutical approach as an anticancer therapeutic strategy for the treatment of BRD9‐dependent tumors. In particular, compound **9** exhibits favorable pharmacokinetic properties and selectively degrades BRD9 over BRD7, addressing one of the key challenges in targeting BRD9. These findings highlight the newly identified degrader as a promising candidate for BRD9 degradation, offering potential to improve therapeutic cancer treatments.

## 4 Experimental Section

### 4.1 Computational Details

#### 4.1.1 Ligand Preparation

Compound **1** was prepared using LigPrep software^[^ ^57^ ^]^ (Schrödinger Suite), accounting for the protonation states at a pH = 7.4 ± 1.0 and minimizing the structure with the OPLS 2005 force field. The related pharmacokinetic properties were also calculated using the Qikprop program in the Schrödinger Suite.^[^ ^58^, ^59^ ^]^

#### 4.1.2 BRD9 Grid Generation and Molecular Docking Experiments

Before performing molecular docking experiments, the Protein Preparation Wizard workflow (Maestro, Schrödinger)^[^ ^60^, ^61^ ^]^ was employed using the crystal structure of the BRD9 bromodomain in complex with BI‐9564 (PDB code: 5F1H).^[^ ^62^ ^]^ All hydrogen atoms were added, and bond orders were assigned. The receptor grid used for docking calculations was generated considering the co‐crystallized ligand, characterized by inner box dimensions of 25 Å and grid center coordinates in Å are X = 4.42, Y = −19.27 and Z = −10.17. Subsequently, docking calculations through Glide software (ligand docking)^[^ ^63^, ^64^, ^65^, ^66^ ^]^ at the XP level were performed to evaluate the docking score values, the interactions with amino acids, and the binding modes.

#### 4.1.3 Pharmacophore Screening

Pharmacophore screening was performed after docking calculations. Therefore, the output docking poses were subjected to pharmacophore screening by applying the developed 3D structure‐based pharmacophore model (“pharm‐druglike2”).^[^ ^36^ ^]^ Using the “Ligand and database screening” tool in Phase,^[^ ^67^, ^68^, ^69^ ^]^ compound **1** was screened, selecting the match of at least 6/7 features and the score in place option.

### 4.2 Chemistry

#### 4.2.1 Synthesis of the Intermediate **1d**

A mixture of 4‐chloro‐1‐ethyl‐[1,2,4]triazolo[4,3‐*a*]quinoxaline (**1c**) (1.0 equiv, 0.205 mmol, 48 mg), obtained following our synthetic protocol reported previously,^[^ ^36^ ^]^ and *m*‐phenylenediamine (3.0 equiv, 0.615 mmol, 67 mg) in DMSO (0.6 mL) was heated under microwave irradiation at 110 °C for 8 minutes. The vial was then cooled to room temperature by air jet cooling, and the reaction mixture was diluted with DCM (1.6 mL) and acidified with 1 N HCl. The resulting precipitate was filtered under reduced pressure, and the obtained solid was collected and precipitated in methanol to give the pure intermediate **1d**, which was then used without further purification (86% yield).

#### 4.2.2 Synthesis of the Intermediate **1f**

To a DMSO solution (1.4 mL) containing 1‐butyl‐4‐chloro‐[1,2,4]triazolo[4,3‐*a*]quinoxaline (**1e**) (1.0 equiv, 0.453 mmol, 118 mg), synthesized as reported previously,^[^ ^37^ ^]^ and *m*‐phenylenediamine (3.0 equiv, 1.359 mmol, 147 mg) was heated under microwave irradiation at 110 °C for 6 minutes. After irradiation, the vial was cooled to room temperature by air jet cooling, and DCM was added to the mixture. The organic phase was washed with water and brine, dried over anhydrous sodium sulfate, filtered, and condensed to afford a crude, which was purified on silica gel column chromatography (from 0% to 2% methanol in DCM) to give compound **1f** (68% yield).

#### 4.2.3 General Synthetic Procedure for the Syntheses of PROTAC Compounds **2**−**9**

To a solution of the intermediate **1d** or **1f** (depending on the final compound) (1.0 equiv, 0.033 mmol) dissolved in anhydrous DMF (0.2 M) was added TEA (5.0 equiv, 0.165 mmol), HATU (1.05 equiv, 0.035 mmol), and the desired carboxylic acid building blocks (linker + E3 ligase ligands) (1.0 equiv, 0.033 mmol). After stirring at room temperature for 1 hour, the mixture was dried over nitrogen flux. The obtained crude was purified by semi‐preparative reversed‐phase HPLC using the binary solvent system (A/B): 0.1% TFA in water (A) and 0.1% TFA in CH_3_CN (B). Specifically, the run was set as follows: gradient conditions from 5% B to 100% B over 50 minutes, a flow rate of 4 mL/min, λ = 240 nm, to obtain the final compounds **2**−**9**, whose purity was determined by HPLC (>96%). All final products were characterized by mass spectrometry experiments and NMR analysis. Detailed structural characterizations, as well as the purity of the tested compounds, are described in the Supporting Information.

#### 4.2.4 In Vitro Alpha Screen Assay

The evaluation of the interaction between synthesized compounds and BRD9 was conducted using AlphaScreen Technology using an Enspire microplate analyzer (Perkin Elmer)^[^ ^70^ ^]^ and the BRD9 (BD1) Inhibitor Screening Assay Kit (BSP‐32519) as reported by Colarusso et al.^[^ ^38^, ^39^ ^]^ IC_50_ values were calculated by GraphPad Prism10 software.

#### 4.2.5 Cell Culture

The AML U937 cell line (ATCC) was grown in Roswell Park Memorial Institute (RPMI) 1640 Medium with the addition of 10% fetal bovine serum (FBS), 1% L‐glutamine, and 1% antibiotics (100 U/mL penicillin, 100 µg/mL streptomycin, and 250 ng/mL amphotericin‐B). Cells have been authenticated by STR profiling.

#### 4.2.6 Western Blotting

Western blot was performed according to the previous protocol.^[^ ^71^, ^72^ ^]^ U937 AML cells were pelleted and then lysed in RIPA buffer (1% Triton X‐100, 0.1% SDS, 150 mM NaCl, 1 mM EDTA pH 8, 10 mM Tris‐HCl pH 8) added with 1% protease inhibitor cocktail. The resulting lysate was subjected to centrifugation for 15 minutes at 4 °C. After that, the supernatant was collected and supplemented with 6X Laemmli buffer (0.217 M Tris‐HCl pH 8.0, 52.17% SDS, 17.4% glycerol, 0.026% bromophenol blue, 8.7% beta‐mercapto‐ethanol) and boiled for 3 minutes at 99 °C,25 µg of protein extract were resolved by SDS‐polyacrylamide gel electrophoresis and then transferred on nitrocellulose membrane (Bio‐Rad). The membrane was probed with anti‐BRD9 (Bethyl Laboratories #A303‐781A), anti‐BRD7 (Invitrogen #PA5‐40379), anti‐GADPH (Elabscience # E‐AB‐40337), and anti‐α‐tubulin (Elabscience # E‐AB‐20036). The protein signal was visualized by enhanced chemiluminescence method (ECL) through the Bio‐Rad Chemidoc machine. ImageJ software was used for densitometric analysis. MG132 (MERCK #M7449) was used to treat U937 cells at the concentration of 25 µM for 6 hours, as previously reported.^[^ ^72^ ^]^

#### 4.2.7 Enhanced Cell Counting Kit 8 (CCK8)

CCK8 was purchased from Elabscience and used according to manufacturer instructions, briefly: 100 µL of cell suspension was added to each well using a 96‐well microplate. Cells were then incubated at 37 °C, in a 5% CO_2_ incubator for 24 hours. Compounds were added to the cells and incubated for an additional 48 hours at 37 °C. Next, 10 µL of CCK‐8 Buffer was added to each well, and cells were incubated again for 4 hours. Next, 450 nm absorbance was measured using a TECAN microplate reader.

#### 4.2.8 Proliferation Assay

Trypan blue exclusion cell counting has been used to trace the number of cells over the different times of analysis.

#### 4.2.9 In Vitro Drug Metabolism Studies

##### Instrumentation

LC‐MS/MS analyses were performed on a Vanquis UHPLC system connected online to a Orbitrap Exploris 120 mass spectrometer (Thermo Fisher Scientific, Bremen, Germany) equipped with a heated electrospray ionization probe (HESI II). All additives and mobile phases were LCMS grade and purchased from Merck (Milan, Italy).

##### Chromatographic Conditions

The chromatographic separation was performed on a Kinetex 2.6 µm Evo C18 100 Å column (100 × 2.1 mm, Phenomenex, Bologna, Italy) using mobile phases A) H₂O and B) ACN, both acidified with 0.1% v/v formic acid. The flow rate and column oven were set at 0.4 mL min^−1^ and 40 °C, respectively. The following gradient was applied: 0.01–8.00 minutes, 5%–95% B, isocratic to 95% B for 1 minute, 9–9.50 minutes, 95%–5% B, followed by 4.5 minutes for column re‐equilibration.

##### Mass Spectrometry Parameters

The ESI was operated in positive mode. The MS was calibrated by Thermo Pierce FlexMix calibration solutions. Full MS parameters: orbitrap resolution: 30000, scan range (m/z): 100–1500, RF lens (%): 70, normalized AGC target (%): 200 maximum injection time (ms): 200. Data‐dependent MS/MS: Orbitrap resolution 15000, isolation window (m/z): 2, collision energy type: normalized, HCD collision energy (%): 30. Ion source parameters: sheath gas pressure: 60 arbitrary units, auxiliary gas flow: 15 arbitrary units, sweep gas: 2 arbitrary units, ion transfer tube temp (°C): 300, vaporizer temp (°C): 300, spray voltage, +3.4 kV, −3.0 kV.

##### Calibration Curve

For the calibration curves, the primary stock solutions (10 mM) were prepared in DMSO. The intermediate stock solutions (0.1 mM) and the working standard solutions were prepared by serial dilution of the stock solutions in methanol to obtain necessary concentrations. Tolbutamide was used as the internal standard (IS, 1 µM). Quantitation of compounds **2** and **9** was performed using linear regression of the response ratios obtained from the calibration curve to calculate the corresponding amount. The method was validated in terms of limits of detection (LOD) and quantification (LOQ) (Table S2). LOD and LOQ were calculated by using the standard deviation (SD) and the slope of the calibration curve, multiplied by 3.3 and 10, respectively.

##### Human Plasma Stability

The plasma stability of compounds **2** and **9** was evaluated. Briefly, the plasma was equilibrated at 37 °C, and biotransformation was initiated by adding the compound solution and mixing. At each specified time point (0, 60, and 120 minutes), test compounds were extracted into 200 µL ice‐cold methanol to stop degradation. Tolbutamide was added during the quenching phase. Finally, the concentration of the test compound was monitored by LC‐MS. The percentage of the test compound remaining at each individual time point was reported. The results are reported as mean ± SD from three independent experiments. Procaine and procainamide were used as controls.

##### Microsomal Stability

The microsomal stability of compounds **2** and **9** was assessed according to the previously described protocol.^[^ ^73^ ^]^ Briefly, 2 µL of each sample were incubated with phosphate buffer (pH 7.4) and human liver microsomes (HLMs, Thermo Fisher Scientific, Bremen, Germany). After pre‐incubation in a water bath for 5 minutes, the mixture was incubated with NADPH at 37 °C in a Thermomixer Comfort (Eppendorf, Hamburg, Germany). The reaction was terminated by adding ice‐cold methanol at different time points (15, 30, 45, and 60 minutes), and the samples were then centrifuged at 10,000 rpm at 25 °C for 5 minutes (Eppendorf microcentrifuge 5424, Hamburg, Germany). The supernatants were collected and analyzed by LC‐MS/MS. The extent of metabolism is quantified as a percentage of the parent compound turnover. The 0‐minute control was prepared by adding the organic solvent immediately after incubation with microsomes. Testosterone was used as a positive control, while the negative control was prepared by incubating the compounds for 60 minutes without cofactor. The results are reported as mean ± SD from three independent experiments and expressed in terms of in vitro microsome half‐lives (t1/2), in vitro intrinsic clearance (CL_int_ *_,_*_in vitro_), and intrinsic in vivo clearance (CL_int_, _in vivo_). In vitro half‐lives (t1/2) were calculated using the expression t1/2 = 0.693/*b*, where *b* is the slope found in the linear fit of the natural logarithm of the fraction remaining of the parent compound versus incubation time. Microsomal intrinsic clearance was calculated as CL_int_ *_,_*_in vitro_  =  (1000) × (0.693/t1/2)/0.5. The intrinsic in vitro clearance was scaled to the intrinsic in vivo clearance (CL_int_, _in vivo_) using a human physiology‐based scaling factor (PBSF): CL_int_ *_,_*_in vivo_ = CL_int_ *_,_*_in vitro_ × PBSF (microsome protein/gram liver: 32 × gram liver/kg b.w.: 25.7)

## Supporting Information

^1^H and ^13^C NMR data and spectra, HRMS data, HPLC chromatograms of final compounds, and Alpha Screen IC_50_ curves of compounds **2** and **9**, in vitro experiments of **dBRD9**, and in vitro pharmacokinetics analysis of compounds **2** and **9** are provided in the Supporting Information.

## Conflict of Interests

The authors declare no conflict of interest.

## Supporting information



Supporting Information

## Data Availability

The data that support the findings of this study are available from the corresponding author upon reasonable request.
