# Supplementary material for: Unveiling New Triazoloquinoxaline‐Based PROTACs Designed for the Selective Degradation of the ncBAF Chromatin Remodeling Subunit BRD9
Source: Chemistry. 2025 May 20;31(34):e202404218. doi: 10.1002/chem.202404218 (PMC12172596; doi:10.1002/chem.202404218)
Supplement: Supplementary file 1 — Supporting Information [file CHEM-31-e202404218-s001.docx]

**Supporting Information**

**An All-in-one Nanohole Array for Size-Exclusive Trapping and High-Throughput Digital Counting of Single Extracellular Vesicles for Non-invasive Cancer Screening**

Lilin Yin^+[a]^, Xianyao Han^+[b]^, Fulin Guo^[a]^, Yuning Zou ^[c]^, Qingpeng Xie*^[d]^, Jianhua Wang^[a]^, Chaoyong Yang*^[c,e]^, Ting Yang*^[a]^

[a] L. Yin, F. Guo, Professor, J. Wang, Professor, T. Yang
Research Center for Analytical Sciences, Department of Chemistry, College of Sciences
Northeastern University
Shenyang, China

[b] X. Han
State Key Laboratory of Synthetical Automation for Process Industries
Northeastern University
Shenyang, China

[c] Y. Zou, Professor, C. Yang

The MOE Key Laboratory of Spectrochemical Analysis & Instrumentation, the Key Laboratory of Chemical Biology of Fujian Province, State Key Laboratory of Physical Chemistry of Solid Surfaces, Collaborative Innovation Centre of Chemistry for Energy Materials, Department of Chemical Biology, College of Chemistry and Chemical Engineering, Xiamen University

Xiamen, China

[d] Q. Xie
Department of Urology
Liaoning Cancer Hospital & Institute, Cancer Hospital of China Medical University
Shenyang, China

[e] Professor, C. Yang
Institute of Molecular Medicine, Department of Gastrointestinal Surgery, Clinical Laboratory, Renji Hospital, Shanghai Jiao Tong University School of Medicine

Shanghai, China

* For correspondence: T. Yang, E-mail: yangting@mail.neu.edu.cn; Q. Xie, E-mail: ly-oscar@outlook.com; C. Yang, E-mail: cyyang@xmu.edu.cn

+ These authors contributed equally: Lilin Yin, Xianyao Han.

**Table of Contents**

**1: Experimental details**

**1.1:** Materials and reagents

**1.2:** Apparatus

**1.3:** Experimental sections

**1.3.1:** Preparation of PNIPAM NPs

**1.3.2:** Preparation of AuNPs

**1.3.3:** Preparation of sEV mimics

**1.3.4:** Cell lines

**1.3.5:** Study design

**1.3.6:** Isolation of sEVs templates using ultra centrifugation method

**1.3.7:** TEM analysis of sEVs

**1.3.8:** Western Blotting (WB) analysis of sEVs

**1.3.9:** Preparation of simulated urine sample

**1.3.10:** Preparation of sEV-imprinted gold nanohole (EI-AuNH) arrays

**1.3.11:** FDTD simulations

**1.3.12:** Calculation of SERS enhancement factor and laser spot diameter

**1.3.13:** The detection of urine sEVs using SEVAK (sEV Analysis Kit)

**1.4:** Smart-Filter to eliminate invalid data

**1.5:** K-Means for spectral clustering

**1.6:** CNN architecture and evaluation metrics

**1.7:** BCa diagnosis model

**1.8:** Feature extraction and visualization

**1.9****:** Statistics and reproducibility

**1.10:** Ethical statement

**2: Supplementary figures and tables**

**Figure S1:** Schematic diagram of the whole synthesis process for EI-AuNH array

**Figure S2:** Representative SEM image of AuNP-AuNH

**Figure S3:** NTA analysis result and TEM image of HV sEVs

**Figure S4:** Western blot result of HV, BCa, PCa and RCa sEVs

**Figure S5:** Representative SEM image of EI-AuNH

**Figure S6:** Representative AFM images of EI-AuNH with different polymerization reaction time and their corresponding PDA imprinting layer thickness

**Figure S7:** NTA analysis result and TEM image of BCa, PCa and RCa sEVs

**Figure S8:** Representative CLSM images of EI-AuNH with various imprinting layer

**Figure S9:** Representative TIRF images of AuNH, AuNP-AuNH, PDA-AuNH, and EI-AuNH capturing DiO-prestained sEVs

**Figure S10:** Schematic representation of the FDTD simulation workspace

**Figure S11:** FDTD simulation of the electromagnetic field distribution in AuNH with different apertures

**Figure S12:** FDTD simulation of electromagnetic field distribution in AuNH with different depth

**Figure S13:** The dependence of the diameter of PNIPAM NP as a function of etching time

**Figure S14:** The dependence of the thickness of gold layer as a function of deposition time

**Figure S15:** Effect of AuNP diameter on SERS enhancement capability

**Figure S16:** Representative Raman spectra of AuNP-AuNHs, AuNHs and AuNPs in 4-MBA and sEV mimics detection

**Figure S17:** Sensitivity validation for single sEV detection and discrimination

**Figure S18:** Representative Raman spectra of EI-AuNHs, AuNP-AuNHs, PDA-AuNHs and AuNHs

**Figure S19:** Smart-Filter used for eliminating invalid data

**Figure S20:** The relationship between sampling step size and valid data percentage

**Figure S21:** The radar plots of the Pearson correlation coefficients between the sEV spectra from the 55 BCa patients and standard spectra

**Figure S22:** The diagnostic performance of all five tested algorithm for the identification of nEVs and taEVs

**Figure S23:** Classification accuracy using different numbers of spectra from each patient

**Figure S24:** Workflow for the diagnosis of BCa based on Method 4

**Figure S25:** The confusion matrix represents the diagnostic results of the test data based on Method 3 and Method 4

**Figure S26:** The Raman intensity at all ten signatures for the identification of nEVs

**Figure S27:** The Raman intensity at all nine signatures for the identification of taEVs

**Table S1:** Information of clinical specimen

**Table S2:** Diagnostic performance of the five diagnostic logics

**Table S3:** Summary of the information of 19 signatures

**1. Experimental details**

**1.1 Materials and reagents**

N-isopropylacrylamide (NIPAM, 98% Aladdin Reagent Co. Ltd. Shanghai, China) was recrystallized by hexane and dried under vacuum for 48 h before use. N, N'- methylenebisacrylamide (BIS), 3-aminopropyltriethoxysilane (APTES), potassium persulfate (KPS), hydrogen peroxide (H_2_O_2_, 30%), dopamine hydrochloride (DA), N-(3-dimethylaminopropyl)-N′-ethylcarbodiimide hydrochloride (EDC), N-hydroxysuccinimide (NHS), 3-aminophenylboronic acid (3-APBA), 4-mercaptobenzoic acid (4-MBA) were purchased from Aladdin Reagent Co. Ltd. (Shanghai, China). Artificial urine was obtained from Leagene Reagent Co. Ltd. (Beijing, China). Gold chloride trihydrate (HAuCl_4_·3H_2_O), hydroxylammonium chloride, DL-1,4-Dithiothreitol (DTT) were the product of Macklin Reagent Co. Ltd. (Shanghai, China). Sodium citrate dihydrate (CIT), ethanol, acetic acid, acetonitrile, Na_2_HPO_4_·12H_2_O, NaH_2_PO_4_·2H_2_O, HCl, sodium hydroxide (NaOH), sulfuric acid (H_2_SO_4_), trimethyl aminomethane (Tris) were obtained from Sinopharm Chemical Reagent Co. Ltd (Shanghai, China). 1,1'-Octadecyl-3,3,3',3'- tetramethylindocyanine perchlorate (DiI), 3,3'-dioctadecyloxacarbocyanine perchlorates (DiO) were provided by Beyotime Biotechnology (Shanghai, China). Ultrafiltration centrifugal tube (100 KDa and 10 KDa MWCO) was obtained from Millipore (Bosten, USA). All the solutions were prepared using deionized water (18 MΩ cm^-1^).

**1.2 Apparatus**

Raman spectra were collected with Horiba XPLORA Raman spectroscopy (HORIBA Scientific, France) and the laser irradiation was performed at 10 mW and 785 nm through a 100× objective lens (NA 0.9). The morphologies of sEVs were observed by a transmission electron microscope (TEM) at an accelerating voltage of 200 kV (JEM2100PLUS, JEOL, Japan). The surface morphologies of the EI-AuNH arrays were observed by a field-emission scanning electron microscope (SEM, SU-8010, Hitachi, Japan). The image of gel electrophoresis was scanned by Tanon 4600SF gel imager (Tanon Science & Technology Co. Ltd.). The sEVs and impurities on the EI-AuNH surface were observed by a total internal reflection fluorescence microscopy (TIRF, Olympus Corporation, Japan) and a FV1200 confocal laser scanning microscope (CLSM, Olympus Corporation, Japan), and the fluorescence was collected under the excitation at 488 nm or 559 nm laser. Nanoparticle tracking analysis (NTA) was carried out by NanoSight NS3000 (Malvern Instrument, England). The size distribution data was collected and analyzed using ZetaView software. Dynamic Light Scattering (DLS) was carried out by Zetasizer Nano S90 (Malvern Instrument, England).

**1.3 Experimental sections**

**1.3.1 Preparation of PNIPAM NPs**

PNIPAM NPs were synthesized through surfactant-free emulsion polymerization. Briefly, 0.54 g N-isopropylacrylamide (NIPAM) and 0.02 g N, N'- methylenebisacrylamide (BIS) were dissolved with stirring in 24 mL deionized water in a three-neck round bottomed flask. After the mixture was heated to 70°C under argon atmosphere, potassium persulfate (KPS, 8 mg mL^-1^) was injected to start the radical polymerization reaction. The reaction was carried out for 4 h at 70°C. The resulting hydrogel microspheres were purified by repeated washing (five times) with deionized water after centrifugation (7000 rpm, 30 min).

**1.3.2 Preparation of AuNPs**

Gold nanoparticles (AuNPs) were prepared by wet-chemical synthetization according to a procedure in literature^[1]^. Briefly, 24 mL of 0.0125% (w/w) gold chloride trihydrate (HAuCl_4_·3H_2_O) was added into a round bottomed flask and then heated to boiling. 1 mL sodium citrate dihydrate solution (1%, w/w) was added into the mixture, stirred and heated to boiling for 15 min. The solution was then cooled down to room temperature and stored in 4℃.

**1.3.3 Preparation of sEV mimics**

sEV mimics were prepared by saturating 100 nm-PNIPAM NPs with 4-mercaptobenzoic acid (4-MBA). The 100 nm-PNIPAM NPs were synthesized according to the procedure described in Section S1.3.1, except that the amount of NIPAM and BIS was 0.27 g and 0.01 g, respectively, and the reaction time was reduced to 2 h. DLS results showed that the diameter of the PNIPAM NPs was 106 nm ± 8 nm. 10 mg of the 100 nm-PNIPAM NPs were then immersed into 1 mL 4-MBA (10^-6^ M) under agitation on a vortex oscillator for 8 h to fill in the hydrogel pore with 4-MBA molecules.

**1.3.4 Cell lines**

Bladder cancer cell lines, including T-24 (Cat. #: CL-0227), 5637 (Cat. #: CL-0002) and Biu-87 (Cat. #: CL-0035) were supplied by Pricella Biotechnology (Wuhan, China). All the cells were cultivated in RPMI-1640 cell culture medium (Pricella, China) supplemented with 10% FBS (Gibco, USA) and 1% antibiotics (Gibco, USA) (5% CO_2_, 95% air, humidity saturation, 37°C).

**1.3.5 Study design**

For evaluating the diagnostic efficacy of the present strategy, a total of 104 participants were enrolled in this study, including 55 bladder cancer (BCa) patients, 11 prostate cancer (PCa) patients, 17 renal cancer (RCa) patients, and 21 healthy volunteers (HV). No self-selection criteria bias for patient populations was anticipated. Morning urine were collected from the participants, sealed and stored at 4℃ for further use. Bloody urine samples were not adopted, as the EVs from blood cells might present interferences to the diagnostic results.

**1.3.6 Isolation of sEV templates by ultracentrifugation**

For the isolation of sEV templates, appr. 500 mL urine sample was collected from healthy volunteer, and subjected to serial centrifugation to remove cells, cellular debris (2,000 g for 20 min) and apoptosis body (60,000 g for 30 min). Afterwards, the urine sample was added with DL-1,4-Dithiothreitol (DTT) and ultracentrifuged at 100,000 g for 60 min at 4°C using a Hitachi Refrigerated Centrifuge (Hitachi, Tokyo, Japan). The low-density sediment was then collected and resuspended in 5 mmol L^-1^ of PBS solution and ultracentrifuged at 100,000 g for 70 min at 4°C again. Finally, the sEV sediments were dispersed in 5 mmol L^-1^ PBS buffer (pH 7.4) and stored at -80°C before further use.

**1.3.7 TEM analysis of sEVs**

sEV suspension was dropped onto a carbon coated copper grid and dried at room temperature for 20 min. Excessive solution was removed with filter paper. Then, 1% phosphotungstic acid was dropped onto the above grid and incubated for 2 min to negatively-stain the sample. Subsequently, the grid was dried under an infrared lamp and observed through TEM at 200 kV.

**1.3.8 Western Blotting (WB) analysis of sEVs**

After identifying the protein concentration by Bicinchoninic acid (BCA) protein assay, 24 μL of each sample was added with 6 μL of 5× loading buffer and heated at 100℃ for 6 min to denature the protein. The proteins were resolved by SDS-PAGE and then transferred onto a polyvinylidene fluoride (PVDF) membrane (Merck Millipore, Immobilon-P Transfer Membrane) through the wet membrane transfer device (Tanon Science & Technology Co., Ltd.). The PVDF membrane was rinsed and blocked with western blocking agent (Dalian Meilun Biotechnology Co., Ltd.) at 37℃ for 2 h. Next, the PVDF membrane was incubated with different antibodies (anti-CD63 antibody, anti-TSG-101 antibody, Proteintech Group, Inc) at 4℃ overnight. After rinsing, the PVDF membrane was incubated with the second antibody (anti-rabbit antibody 1:5000; anti-mouse antibody 1:10000, Proteintech Group, Inc) at 37℃ for 2 h. Finally, the blots were presented through Tanon 4600SF Imaging System (Tanon Science & Technology Co. Ltd.).

**1.3.9 Preparation of simulated urine sample**

Urine samples (100 mL) were collected from a mixture of several bladder cancer patients to simulate the real interfering impurities typically present in patient urine matrices. The urine sample was centrifuged at 50,000 g for 30 min. The resulting precipitates (i.e., larger impurities) and the supernatant was collected separately. The supernatant was then subjected to the sEV isolation step, from which sEVs and sEV-free urine were collected separately.

Subsequently, the sEV-free urine was purified by ultrafiltration (10 KDa) to collect small impurities. The large and small impurities were then mixed and stained with DiI (10 μM) for 20 min, followed by purification via ultrafiltration (10 KDa) and resuspended in 100 μL PBS buffer (5 mmol L^-1^, pH 7.4). Meanwhile, 100 μL BCa sEV suspension (~10^11^ particles) was mixed with 1 mL DiO (10 μM) solution for 20 min-staining. Afterwards, the stained sEVs were purified by ultrafiltration (100 KDa) and resuspended in 100 μL PBS buffer (5 mmol L^-1^, pH 7.4). Finally, 100 μL of DiI-stained urine impurities and an equal volume of DiO-stained sEVs were mixed with 100 mL artificial urine (sEV-free, Leagene, China) for the evaluation of sEV isolation performance.

**1.3.10** **Preparation of sEV-imprinted gold nanohole (EI-AuNH) arrays**

We first prepared highly ordered PNIPAM NPs arrays using gas-liquid interface self-assembly method^[2]^. Cover glass was first immersed in a mixture of H_2_SO_4_ /H_2_O_2_ (7: 3, v/v) for 12 h followed by repeated rinsing with deionized water. The glass slide was dried in a stream of N_2_, and dropped with 10 μL of the PNIPAM NP suspensions and 50 μL of ethanol. After drying in N_2_ stream at room temperature, a quasi-hexagonal 2D array of PNIPAM NP was formed on top of the glass slide. The PNIPAM NPs arrays were placed in a plasma cleaner, where the PNIPAM NPs were etched for 90 s.

The glass slide harboring PNIPAM arrays were then functionalized with 3-aminopropyltriethoxysilane (APTES) by vapor phase deposition^[2]^. Briefly, the glass slide was immersed with APTES solution at an atmosphere pressure of 0.3 mbar in a desiccator for 30 min, and heated at 80°C for 1 h. A total of 100 μL AuNP suspension was added on top of the glass slide for seeding AuNPs through electrostatic interaction. After 4 h-incubation, the glass slide was rinsed repeatedly with deionized water. The PNIPAM NPs were then removed by ultrasonication in a H_2_O: CH_3_OH (1: 20, v/v) mixture for 5 min. Afterwards, the glass slide was incubated with 3 mL of gold growing solution containing 0.4 mM hydroxylamine hydrochloride and 0.5% (w/v) HAuCl_4_·3H_2_O for 2 h under agitation on a vortex oscillator for in-situ growth of a gold layer along with AuNP seeding area, generating a gold nanohole (AuNH) array. The AuNH array was rinsed with deionized water and dried in a N_2_ stream for further use.

The AuNH arrays were incubated with AuNP suspensions for 4 h at room temperature to deposit in the nanoholes through electrostatic interaction with amine groups on the glass slide surface. 3-Aminophenylboronic acid (3-APBA) were then decorated on the surface of AuNPs in the nanoholes through EDC/NHS coupling. As gold layer outside of the nanoholes are grown by NH_2_OH·HCl reduction method, only AuNPs in the nanoholes possess citrate groups that can react with 3-APBA. To be specific, the glass slide was incubated with a mixture of 1 mL 3-APBA (6 mg mL^–1^) solution, 1 mL of EDC (50 mg mL^–1^) and 1 mL of NHS (50 mg mL^–1^) for 60 min-conjugation at room temperature. This resulted in the generation of AuNP-AuNH arrays. After repeated washing with PBS buffer (5 mmol L^-1^, pH 7.4), the AuNP-AuNH arrays were incubated with 1 mL of blocking buffer (10 mM PBS, 1% BSA, pH 7.4) for 30 min at 37°C to block the nonspecific binding sites.

For sEV imprinting, 10 μL of the templating sEVs were added on top of the AuNP-AuNH arrays at 4°C for 4 h to anchor the template sEVs. Afterwards, the sEV-coated surface was immersed into 10 mL of Tris-HCl buffer (10mM, pH 8.5) containing 20 mg of dopamine hydrochloride. The sEV imprinting polydopamine (PDA) layer gradually formed after 12 h polymerization under stirring. The arrays were then immersed into PBS buffer (5 mmol L^-1^, pH 7.4) for a 10-second ultrasonic treatment, followed by incubation with 1 mL of blocking buffer (10 mM PBS, 1% BSA, pH 7.4) for 30 min at 37°C to block the nonspecific binding sites. After treating with CH₃COOH:  C₂H₃N (2: 98, v/v) mixture for 30 min to remove sEV templates, sEV-imprinted gold nanohole (EI-AuNH) array was formed. The resulting (EI-AuNH) was immersed in PBS solution at 4°C for further use.

**1.3.11 FDTD simulations**

Three-dimensional (3D) finite difference time domain (FDTD) simulations were performed to simulate the electromagnetic field distribution of the different structures^[3,4]^. The electromagnetic field distribution of bare AuNHs and AuNP-AuNHs were simulated under a 785 nm laser excitation with a mesh size of 2 nm. Symmetric/anti-symmetric boundary conditions (BCs) set along *x* and *y* direction extend the plasmonic response over an infinite 2D array while reduce the simulation time by a factor of 8 without worsening the accuracy of the results. The AuNPs were modeled as homogeneous gold spheres, while the bare substrate was represented as a thick dielectric layer of glass. The laser was irradiated along the normal direction of the AuNH arrays surface. The far-field electromagnetic field distributions were monitored 1 nm away from the top of the nanohole structures. The electromagnetic field distributions in the nanoholes were monitored half of the depth away of the nanohole bottom. The near-field electromagnetic field distributions within the X-Z plane were obtained within the top layer of the AuNH array structures.

**1.3.12 Calculation of SERS enhancement factor and laser spot diameter**

In order to evaluate the SERS effect of the AuNHs structures, the SERS enhancement factor (EF) was calculated according to the following equation

$EF=\frac{I_{SERS}/C_{SERS}}{I_{RS}/C_{RS}}$ ()

where *C_SERS_* and *C_RS_* refer to the concentration of analytes at the SERS detection mode and the normal Raman spectroscopy (RS) detection mode, *I_SERS_* and *I_RS_* refer to the corresponding signal intensity. The Raman spectra were measured under 785 nm laser excitation (Horiba XPLORA) through a 100× objective, with the laser power of about 15 mW and the integration time of 10 s. 10 μL of 4-mercaptobenzoic acid (4-MBA, *C_RS_* = 10 mM) solution was dropped and spread on the surface of the glass slide to measure *I_RS_* which was derived to be 27.8 at 1069 cm^-1^. In the SERS mode, taking AuNP-AuNH array for example, signals were detected at a lower concentration (*C_SERS_* = 1 μM) and *I_SERS_* was measured to be 1179. Accordingly, the EF of AuNP-AuNH was calculated to be 3.28 × 10^4^. The EF factor of AuNH array and AuNPs were obtained similarly.

In order to evaluate the spatial Raman enhancing ability of AuNP-AuNH arrays, 10 μL of sEV mimics, i.e., PNIPAM NPs saturated with 10^-3^ M 4-MBA was dropped and spread on the surface of AuNP-AuNH arrays, and dried at room temperature. The Raman spectra were measured under a 785 nm laser excitation (Horiba) through a 100× objective with a laser power of about 15 mW and the integration time of 10 s. The spatial Raman enhancing ability of AuNH array and AuNPs were evaluated similarly.

The laser spot diameter was calculated according to the following equation

$D=1.22\times\frac{\lambda}{NA}$ ()

where *λ* is the laser wavelength (785 nm) and *NA* is the numerical aperture of objective lens (0.9). Accordingly, the laser spot diameter was calculated to be 1064 nm.

**1.3.13 The detection of urine sEVs using SEVAK (sEV Analysis Kit)**

The patient's urine samples were collected and adjusted to pH 8.5. Afterwards, 2 mL of the urine samples were injected into the inlet of SEVAK by a sterile syringe. The SEVAK device was placed with the inlet upward to allow samples flowed through the EI-AuNH array. It took about 20 min for 2 mL urine to completely flow through. Afterwards, 2 mL PBS buffer (10 mM pH 8.5) was injected to wash away non-specifically adsorbed impurities on the surface of EI-AuNH array. After sEV capture, the Raman spectrum of EI-AuNH array were collected through the detection window. The sEV capture ability presented in Fig. 2d was indicated by the mean fluorescence intensity of sEV per unit area, i.e., the average fluorescence density of capture sEVs shown in Fig. 2b, whereas the capture ability, purity (%) and co-localization ratio (%) presented in Fig. 2d were calculated according to the following equation

$Capture\text{ }ability=I_{sEV}=\frac{Int_{Green}}{Area}$ ()

$Purity(\%)=\frac{I_{sEV}}{I_{sEV}+I_{impurity}}\times100\%$ ()

$Co-localization(\%)=\frac{I_{sEVih}}{I_{sEV}}\times100\%$ ()

where *Int_Green_* refers to the green fluorescence, *Area* refers to the area of material within the field of view, *I_sEV_* refers to the mean fluorescence intensity of sEV, *I_impurity_* refers to the mean fluorescence intensity of impurities and *I_sEVih_* refers to the mean fluorescence intensity of the sEVs in the nano hole of EI-AuNH.

**1.4 Smart-Filter to eliminate invalid data**

Smart-Filter was used to eliminate the invalid spectral data arising from spots containing partial sEVs or the nanohole exterior. Raman spectra from 289 complete, individual nanoholes were collected and an average Raman intensity at 998 cm^-1^ (*I*998 cm^-1^ = 3700±300) was used as a benchmark to identify the invalid spectral data. Those data with *I*998 cm^-1^ < 3400 were identified as invalid data, which were eliminated through an automatic screening program written in Python.

**1.5 K-Means for spectral clustering**

Urine samples from 21 HVs and 55 BCa patients were processed with SEVAKs for sEV isolation and SERS detection. For each sample, 200~300 sets of single sEV spectra were collected and underwent Smart-Filter to eliminate invalid data. Before spectral clustering using K-Means, all the valid data were normalized using Raman signal at 998 cm^-1^ as internal standard and then underwent feature pre-screening by eliminating irrelevant data originating from the substrate or internal standard (400-427 cm^-1^, 503 -553 cm^-1^ and 966-1009 cm^-1^).

For sEV spectral classification, a total of 1100 spectra from empty nanoholes were assembled to define blank spectra (Std. blank). To establish criteria defining typical tEV spectra, we obtained sEVs secreted from three independent BCa cell lines, representing the most prevalent clinical BCa types (T24, BIU-87, and 5637 cells). In total, 1623 spectra from six batches of culture medium (two per cell line) were used for tEV identification. We divided the spectra collected from these BCa sEVs into two clusters using K-Means and then compared the Pearson correlation coefficients (*r*) between the spectra of sEVs from these BCa sEVs and blank spectra from empty nanoholes. Those spectra that has relatively lower *r* value were identified as “tEV” spectra and labeled as “Std. tEV”. Similarly, clustering of the spectra from the 21 healthy volunteers and further comparison of correlation coefficients between sEV spectra from these healthy volunteers and blank spectra resulted in the identification of “Std. nEV”.

The spectra from the 55 BCa patients were clustered into three groups, and the *r* values between each sEV spectrum and the standard spectra (Std. blank, Std. tEV, Std. nEV) were subsequently compared. Spectra exhibiting greater similarity (i.e., higher *r* value) with the Std. tEV spectra than with the Std. nEV or Std. blank spectra were classified as taEV spectra. Spectra showing higher similarity with the Std. nEV spectra were identified as nEV spectra, while those more closely resembling the Std. blank spectra were identified as blank spectra (Figure S21). In this way, the sEV spectra from all the 55 BCa patients can be classified into three categories, i.e., blank, nEV and taEV spectra.

**1.6 CNN architecture and evaluation metrics**

After labeling sEV spectra into three categories, a convolutional neural network (CNN) model was further constructed for sEV spectra classification. The CNN architecture was determined using a grid search algorithm. The initial two convolutional layers employed filters of varying sizes: 32 filters with a (3, 5) kernel size and 64 filters with a (2, 2) kernel size. After passing through a max-pooling layer, we connected to a third convolutional layer that employed 128 filters with a kernel size of (2, 2). All convolutional operations utilized a stride of (1, 1). The resulting features were then flattened and inputted into a fully connected layer containing 100 neurons. To mitigate overfitting, a Dropout layer with a 50% dropout rate was added. Finally, a fully connected layer with 3 neurons was used to produce the final scores for each class label. These scores were then passed through a SoftMax activation function to compute the final class probabilities. The complete model comprised 1,654,867 trainable parameters.

To address class imbalance issues within the training data, we implemented class weights during model training, enhancing the model's classification accuracy while improving overall model robustness.

The CNN model's performance was evaluated using the following metrics:

1) Classification accuracy: The proportion of all samples correctly classified by the model

$Accuracy=\frac{TP+TN}{TP+TN+FP+FN}$ ()

where *TP* is the number of correctly classified positive samples, *TN* is the number of correctly classified negative samples, *FP* is the number of negative samples incorrectly classified as positive, and *FN* is the number of positive samples incorrectly classified as negative.

2) Sensitivity: The model's ability to identify all positive samples, representing the proportion of actual positive cases correctly identified.

$Sensitivity=\frac{TP}{TP+FN}$ ()

3) Precision: The proportion of positive samples correctly classified as positive by the model.

$Precision=\frac{TP}{TP+FP}$ ()

**1.7 BCa diagnosis model**

By using the proposed CNN model and a digital counting system, we developed a diagnostic system for BCa patients. The detailed workflow is shown in Fig. 6a. Briefly, 170 valid spectra were collected for each patient and processed through the trained CNN model. The model output the predicted origin of each spectrum, resulting in a digital counting matrix comprising the number of blank, nEV, and taEV spectra. A series set of sEV digital counting matrix for all the 76 participants (healthy volunteer and BCa patients) was thus obtained. The count of blank spectra (*ε*_1_), nEV spectra (*ε*_2_), and taEV (*ε*_3_) were thereby derived, with the count of total sEV calculated as [*ξ*_1_ = *ε*_2_ + *ε*_3_] and the proportion of taEVs within total sEVs as [*ξ*_2_ = *ε*_3_ / *ξ*_1_]. All thresholds are based on training data statistics and determined. First, a threshold (*δ*_1_) of 53 was set (the third quartile *ξ*_1_ for healthy samples). If *ξ*_1_ < *δ*_1_, the individual was classified as healthy. Otherwise, the individual proceeded to the next discriminator. Then, a threshold (*δ*_2_) of 10 was set (determined based on the lower limit after removing outliers from earlier patients’ *ε*_3_). If *ε*_3_ < *δ*_2_, the individual was considered as non-cancerous. Otherwise, the individual was diagnosed as a BCa patient and entered next discriminator. Finally, a threshold (*δ*_3_) of 0.38 was set (the third quartile of early-stage BCa patient samples’ *ξ*_2_). If *ξ*_2_ < *δ*_3_, the individual was considered as early-stage BCa patient. Otherwise, the individual was diagnosed as a terminal-stage BCa patient.

Method 1 (based on total sEV count, ξ1) for comparison is described in the following discriminant logic. A threshold (*δ*_1-1_) of 53 (3rd quartile of healthy samples’ *ξ*_1_) and a threshold (*δ*_1-2_) of 82 (3rd quartile of early-stage samples’*ξ*_1_) were set. If *ξ*_1_ < *δ*_1-1_, the individual was considered as healthy. If *δ*_1-1_<*ξ*_1_ < *δ*_1-2_ the individual was diagnosed as an early-stage BCa patient. Otherwise, the individual was diagnosed as a terminal-stage BCa patient.

Method 2, based on taEV count (ε_3_), follows the discriminant logic outlined below. A threshold (*δ*_2-1_) of 10 representing the minimum value after removing outliers of early-stage samples’ *ε*_3_, and a threshold (*δ*_2-2_) of 25 representing the minimum value after removing outliers of terminal-stage samples’ *ε*_3_ were set. If *ε*_3_ < *δ*_2-1_, the individual was classified as healthy. If *δ*_2-1_<*ε*_3_ < *δ*_2-2_, the individual was classified as an early-stage BCa patient. Otherwise, the individual was diagnosed as a terminal-stage BCa patient.

Method 3, based on the proportion of taEVs within total sEVs (ξ_2_), for comparison, is described in the following discriminant logic. A threshold (*δ*_3-1_) of 0.146 (3rd quartile of healthy samples’ *ξ*_2_) and a threshold (*δ*_3-2_) of 0.378 (3rd quartile of early-stage samples’*ξ*_2_) were set. If *ξ*_2_ < *δ*_3-1_, the individual was considered as healthy. If *δ*_3-1_<*ξ*_2_ < *δ*_3-2_, the individual was diagnosed as an early-stage BCa patient. Otherwise, the individual was diagnosed as a terminal-stage BCa patient.

Method 4, based on total sEV count (ξ_1_) and taEV count (ε_3_), follows the discriminant logic outlined below. A threshold (*δ*_4-1_) of 53 representing the 3rd quartile of healthy samples’ *ξ*_1_, and a threshold (*δ*_4-2_) of 25 representing the minimum value after removing outliers of terminal-stage samples’ *ε*_3_ were set. If ξ_1_ < *δ*_4-1_, the individual was classified as healthy. Then, a threshold (*δ*_4-2_) of 25 was set (determined based on the lower limit after removing outliers from terminal-stage samples’ *ε*_3_). If *ε*_3_ < *δ*_4-2_, the individual was considered as early-stage samples. Otherwise, the individual was diagnosed as terminal-stage samples.

**1.8 Feature extraction and visualization**

Gradient-weighted Class Activation Mapping (Grad-CAM) is a widely used method for visualizing the decisions of CNNs. It highlights the key regions in an image that contribute most to a given classification result by generating class-specific heatmaps. In this study, we utilized Grad-CAM to understand the model's decision-making process for sEV classification. To develop Grad-CAM, we computed the gradient of the output class with respect to the feature maps of the last convolutional layer, thereby identifying the contribution of each location in the feature map to the final prediction. These computed gradients were then subjected to Global Average Pooling (GAP) to derive the importance weights for each feature map, representing their significance for the specific class. Finally, each filter in the final convolutional layer was multiplied by its corresponding importance weight. The weighted filters were summed and passed through a ReLU activation function to generate a Grad-CAM heatmap. The corresponding calculation formula is as follows:

$\text{L}_{\text{Grad-CAM}}^{\text{c}}=\mathrm{Re}\text{LU}(\sum_{k} \alpha_{k}^{c}A^{k})$ ()

where *A* is the feature layer output by the last convolutional layer, *k* represents the *k*^th^ channel in the feature layer *A*, *c* represents category, *A^k^* represents the data of channel *k* in feature layer *A*, $\alpha_{k}^{c}$ represents the weight for *A^k^*. The calculation formula for $\alpha_{k}^{c}$ is as follows:

$\alpha_{k}^{c}=\frac{1}{z}\sum_{i} \sum_{j} \frac{\partial y^{c}}{\partial A_{ij}^{k}}$ ()

where *y^c^* represents the score predicted by the network for category *c*, $A_{ij}^{k}$represents the data of feature layer *A* in channel *k* at coordinates (*i*, *j*), and *z* represents the product of the width and height of the feature layer^[5]^. This heatmap was overlaid on the original sEV spectra, offering a clear visual representation of the regions that influenced the model's decision. Grad-CAM analysis of the CNN classification results pinpointed the most discriminative features in nEVs and taEVs.

According to the Grad-cam interpretation, the top 3% of features in both nEVs and taEVs were identified, including 10 features in nEVs and 9 features in taEVs. Subsequently, these features were traced back to their corresponding peak positions in the original Raman spectra data, revealing the specific spectral bands that influenced the classification results.

To further explore the distribution of features across different cancer types, the taEV features were investigated. Data were extracted from healthy volunteers, BCa patients, as well as patients with RCa and PCa. For effective visualization of the high-dimensional taEV features with 9 identified features, principal component analysis (PCA) was adopted for dimensionality reduction. PCA successfully reduced the data to a 3-dimensional space, capturing a high explained variance ratio of 98.40%. This indicates that the key features were retained during dimensionality reduction. This transformation enabled us to visualize the data in 3D space, providing valuable insights into the distribution of the identified features across different cancer types.

**1.9 Statistics and reproducibility**

All data processing (including Smart-Filter, K-Means, CNN, Grad-CAM, and PCA) was handled through custom Python code. Python 3.7.6 with TensorFlow 2.5.0, Keras, scikit-learn, scipy, numpy, and pandas were used for model training and testing. All code was developed using open-source tools and packages.

The statistical analysis (e.g., t test) was performed using Python scipy. The differences were considered significant at p<0.05 (**p < 0.01, ***p <0.001, ****p < 0.0001), and considered to have no significance at p>0.05 (ns). All code was developed using open-source tools and packages. All visualizations of data were made using Origin software (version 2021), Python and MATLAB R2021a (MathWorks) codes. All experiments were repeated independently with similar results for at least three times, especially micrograph results.

**1.10** **Ethical statement**

All the participants were recruited from Liaoning Cancer Hospital. Only patients with definite information of sex, age, and pathological diagnosis were recruited. The study complied with all the relevant ethical regulations and was approved by the Ethics Committee of Northeastern University, China (No. NEU-EC-2023B013S). Informed, written consent was obtained from either the participant or next of kin by the co-author (Q.P.X.).

**2. Supplementary figures and tables**

**Figure S1.** Schematic diagram of the whole synthesis process for EI-AuNH array. Poly-N-isopropylacrylamide nanoparticle (PNIPAM NP) suspension was first dropped on the surface of a glass slide (1). Ethanol was then added and allowed to evaporate, resulting in the formation of a quasi-hexagonal PNIPAM array (2). After amine functionalization by vapor phase deposition of APETS, AuNPs were deposited onto the glass slide, generating PNIPAM&AuNPs (3). The PNIPAM templates were removed by ultrasonication, forming AuNP array (4). After in situ reduction of HAuCl_4_, AuNH array was formed (5). AuNPs inside the nanoholes were decorated with APBA by EDC/NHS coupling, resulting in AuNP-AuNH array (6). sEV templates were immobilized on the surface of AuNP-AuNH array (7), followed by the coating of PDA layer for sEV imprinting (8). After removing the sEV templates, EI-AuNH array was finally formed (9).

**Figure S2.** Representative SEM image of the AuNP-AuNH. Arrow indicates AuNP. Scale bar: 500 nm.

**Figure S3.** NTA analysis result of sEVs isolated from healthy volunteers by ultracentrifugation and their representative TEM image. Scale bar: 100 nm.

**Figure S4.** Western blot result of sEV isolated from healthy volunteer (HV), bladder cancer patient (BCa), prostate cancer patient (PCa) and renal cancer patient (RCa). CD63 and TSG101 are common sEV markers.

**Figure S5.** Representative SEM image of EI-AuNH. Yellow arrow indicates AuNP, while red arrow indicates the imprinting layer. Scale bar: 500 nm.

**Figure S6.** (a) Representative AFM images of EI-AuNH with different polymerization reaction time. (b) Corresponding PDA imprinting layer thickness and the total thickness of the array as a function of reaction time. (n = 3 measurement results for 3 different positions, mean ± SD).

**Figure S7.** NTA analysis results of sEVs isolated from BCa patient (a), PCa patient (c) and RCa patient (e) and their representative TEM images (b, d, f). Scale bar: 100 nm.

**Figure S8.** (a) Representative CLSM images of EI-AuNH with various imprinting layer thickness capturing 1×10^9^ particles of DiO-prestained sEVs. Scale bar: 25 μm. (b) The sEV capture capability as a function of imprinting layer thickness. sEV capture capability is represented by the fluorescent optical density of the fluorescence emitted by DiO (n = 5 measurement results for different positions, mean ± SD).

**Figure S9.** Representative TIRF images (merged with bright field images) of AuNH (a), AuNP-AuNH (b), PDA-AuNH (c), and EI-AuNH (d) capturing 9.7×10^8^ particles of DiO-prestained sEVs. Scale bar: 20 μm. The white circle indicates sEVs captured inside the nanoholes, whereas the red circle indicated those outside the nanoholes.

**Figure S10.** Schematic representation of the FDTD simulation workspace

**Figure S11.** FDTD simulation of the electromagnetic field distribution in AuNH with different apertures.

**Figure S12.** FDTD simulation of electromagnetic field distribution in AuNH with different depth.

**Figure S13.** (a) Representative SEM images of the PNIPAM NP arrays with various etching time. Scale bar: 5 μm. (b) The diameter of PNIPAM NP as a function of etching time. For the fabrication of 220 nm AuNHs, 90 s was chosen as the optimal etching time (n = 10 PNIPAM NPs, mean ± SD).

**Figure S14.** (a) Representative SEM images of the AuNH arrays with various gold deposition time. (b) The thickness of the gold layer and the total thickness as a function of deposition time. For the fabrication of AuNH with 75 nm depth, 2 h was chosen for gold deposition (n = 3 measurement results for 3 different positions, mean ± SD).

**Figure S15.** Effect of AuNP diameter on SERS enhancement capability. (a) Raman intensity at 1076 cm^-1^ and (b) representative Raman spectra of 4-MBA acquired using EI-AuNH arrays with AuNPs of various diameters as SERS substrates.

AuNPs with different diameters (~15 – 40 nm) were prepared by adjusting the volume of gold chloride trihydrate added during the synthesis process and used for the preparation of EI-AuNH array. The SERS enhancement capability of these EI-AuNH arrays was further evaluated using 4-MBA (10^-6^ M) as the Raman reporter molecule. As shown in Figure S15, the Raman intensity at 1076 cm^-1^ significantly increases as the diameter of AuNPs increases from 17 to 29 nm, but then gradually increases when the diameter exceeds 29 nm. Larger AuNPs generally offer stronger SERS enhancement due to their increased electromagnetic field strength. However, as the particle size increases, fewer AuNPs can fit into each nanohole of the array and less hotspot forms, resulting in a less increment in the overall Raman intensity. Importantly, as larger AuNPs are unstable and may potentially lead to uneven signals, we finally selected AuNPs with a diameter of approximately 29 nm as the optimal choice.

**Figure S16.** Representative Raman spectra of AuNP-AuNHs, AuNHs and AuNPs in 4-MBA and sEV mimics detection.

For the detection of 4-MBA molecules, AuNP-AuNH gave rise to 5.54 times higher signal (*I*1076 cm^-1^) compared to AuNPs, whereas AuNH gave rise to 2.57 times higher signal (*I*1076 cm^-1^) compared to AuNPs. In the case of sEV mimics, the Raman intensity at 1076 cm^-1^ enhanced by AuNP-AuNH was 17.95 times higher than that by AuNPs, and the Raman intensity at 1076 cm^-1^ enhanced by AuNH was 8.81 times higher than that by AuNPs. This discrepancy highlighted the spatial SERS enhancing capability of AuNP-AuNH and demonstrated that AuNP-AuNH is more efficient for three dimensional targets.

**Figure S17.** (a) Representative Raman spectra of a normal sEV (nEV) or tumor-associated sEV (taEV) from the same BCa patient (n=35 replicates for Raman spectra of EI-AuNH with/without nEV or taEV). Line represents mean, shading indicates s.d. (b) The corresponding average Raman intensities at 589 cm^-1^, 620 cm^-1^, 746 cm^-1^ and 1120 cm^-1^, respectively. Unpaired two-sided Student’s t test. The green dash line indicates three times the standard deviation of the blank signal. *p < 0.05, **p < 0.01, ***p < 0.001, ****p < 0.0001.

For single sEV detection and discrimination, our method must be sensitive enough to produce Raman signals from individual sEVs that are not only distinguishable from background fluctuations, but also differentiable between nEVs and taEVs.

To evaluate whether our method meets this requirement, we conducted two key analyses: (1) Blank vs. Signal comparison: For a given BCa patient, we collected 221 sets of Raman spectra, including 123 blank spectra (from empty nanoholes), 58 spectra from nEVs, and 40 spectra from taEVs. For fair comparison, 35 sets of spectra for each type (blank, nEV and taEV) were randomly selected and used for the following analysis. We selected specific wavenumbers corresponding to four established BCa Raman signature peaks (Fig. 7b). At each wavenumber, we calculated the background fluctuation by determining three times the standard deviation of signals from blank nanoholes (indicated by the green dashed lines in Figure S17b). Raman signals from both nEVs and taEVs were then compared to these thresholds. As shown in Figure S17b, both types of sEV signals clearly exceed background fluctuation levels, confirming that our method is sensitive enough to detect single sEVs. (2) nEV *vs.* taEV differentiation: We further conducted statistical significance testing (*P*-value analysis) to assess whether Raman signals from nEVs and taEVs were significantly different. The results confirmed a statistically significant difference, which forms the basis for downstream data processing and classification using machine learning models, and ultimately supports the feasibility of our method for BCa diagnosis.

**Figure S18.** Representative Raman spectra of EI-AuNHs, AuNP-AuNHs, PDA-AuNHs and AuNHs. By comparing the spectra between EI-AuNHs and AuNP-AuNHs, or between PDA-AuNHs and AuNHs, it is concluded that PDA layer ultimately had no discernable impact on the degree of enhancement by AuNHs.

**Figure S19.** Smart-Filter used for eliminating invalid data. (a) Working principle for Smart-Filter. Raman spectral data arising from spotted areas containing partial sEVs or the nanohole exterior were regarded as invalid data, while those from individual sEVs within a nanohole were regarded as valid data. (b) Average Raman intensity at 998 cm^-1^ of Raman spectra collected from 289 complete, individual nanoholes. (c) The number of Raman spectra collected from all the 55 BCa patients before and after data filtration, showing 10~15% of the data were invalid and were eliminated by Smart-Filter.

The average distance between two adjacent nanoholes in EI-AuNH array was determined to be 1016±105 nm from its SEM images, and the average diameter of the nanoholes was 230±21 nm. Consider the laser beam diameter of ~ 1 μm, there is a possibility that the beam may simultaneously cover two adjacent nanoholes (both partially spotted), an incomplete nanohole, or even a non-nanohole region. These scenarios are considered invalid, as the acquired signals do not originate from a single, fully exposed nanohole containing one sEV. To address this, we applied a Smart-Filter algorithm to identify and eliminate such invalid measurements. Considering APBA was only present in the nanohole interior, its Raman signal was used as an internal standard to assess the validity of each spectrum. If the laser beam spots two nanoholes at a time, or spots incomplete nanohole, or even non-nanohole area, the APBA signal would be less than collected from the whole nanohole. Base on this principle, those invalid measurement can be filtered out and eliminated.

**Figure S20.** The percentage of valid data (a) and the percentage of valid sEV data accounted for all valid spectra (b) as a function of laser step size, showing that 5 μm is the optimal step size to get maximum percentage of valid data. n = 3 samples for (a), and 5 samples for (b), mean ± SD

**Figure S21.** The radar plots of the Pearson correlation coefficients (*r*) between the sEV spectra from the 55 BCa patients and standard spectra (“Std. blank”, “Std. nEV” or “Std. tEV”). (a) the *r* value between Std. blank spectra and blank, nEV or taEV spectra of the 55 BCa patients, indicating a relatively higher correlation (or similarity) between Std. blank spectra and blank spectra of the BCa patients. (b) the *r* value between Std. nEV and the nEV or taEV spectra of the 55 BCa patients, indicating a relatively higher correlation (or similarity) between Std. nEV and nEV spectra of the BCa patients. (c) the *r* value between Std. tEV and the nEV or taEV spectra of the 55 BCa patients, indicating a relatively higher correlation (or similarity) between Std. tEV and taEV spectra of the BCa patients.

**Figure S22.** The Radar plot indicating the total accuracy of all five tested algorithms, including SVM, LSTM, Naive Bayes, Decision Tree and the present strategy.

**Figure S23.** Classification accuracy using different numbers of spectra from each patient (n=10-200, mean ± SD).

**Figure S24.** Workflow for the diagnosis of BCa based on Method 4, i.e., the total sEV count (ξ_1_) and taEV count (ε_3_). Threshold δ_1_ = 53 (the third quartile *ξ*_1_ for healthy samples), threshold δ_2_ = 28 (the third quartile ε_3_ for early-stage BCa patient samples)

**Figure S25.** The confusion matrix represents the diagnostic results of the test data based on (a) Method 3, i.e., the proportion of taEVs within total sEVs (ξ_2_), and based on Method 4, i.e., total sEV count (ξ_1_) and taEV count (ε_3_). HV, healthy volunteer; ES, early-stage patients; TS: terminal-stage patients.

**Figure S26.** The Raman intensity at all ten signatures for the identification of nEVs (n=21 for control, 55 for nEV or taEV, 6 for cell, mean ± SD).

**Figure S27.** The Raman intensity at all nine signatures for the identification of taEVs (n=21 for control, 55 for nEV or taEV, 6 for cell, mean ± SD).

Table S1. Information of clinical specimen

| Specimen No. | Sex | Age | Clinical diagnosis | |
| --- | --- | --- | --- | --- |
|  |  |  | Cancer type | Cancer stage |
| 1 | Male | 49 | BCa | TaN0M0 |
| 2 | Male | 55 | BCa | T1N0M0 |
| 3 | Male | 63 | BCa | T1N0M0 |
| 4 | Female | 66 | BCa | T1N0M0 |
| 5 | Female | 66 | BCa | TaN0M0 |
| 6 | Female | 60 | BCa | TaN0M0 |
| 7 | Male | 59 | BCa | TaN0M0 |
| 8 | Male | 58 | BCa | T1N0M0 |
| 9 | Female | 74 | BCa | T4N0M0 |
| 10 | Female | 71 | BCa | T4N0M0 |
| 11 | Male | 75 | BCa | T2N0M0 |
| 12 | Male | 70 | BCa | T1N0M0 |
| 13 | Male | 70 | BCa | T1N0M0 |
| 14 | Female | 69 | BCa | TaN0M0 |
| 15 | Female | 77 | BCa | T3N2M0 |
| 16 | Male | 61 | BCa | T1N0M0 |
| 17 | Female | 58 | BCa | T1N0M0 |
| 18 | Male | 53 | BCa | TaN0M0 |
| 19 | Female | 62 | BCa | T3N0M0 |
| 20 | Male | 67 | BCa | TaN0M0 |
| 21 | Male | 51 | BCa | TaN0M0 |
| 22 | Male | 77 | BCa | TaN0M0 |
| 23 | Male | 64 | BCa | TaN0M0 |
| 24 | Male | 75 | BCa | T1N0M0 |
| 25 | Female | 82 | BCa | T1N0M0 |
| 26 | Male | 50 | BCa | TaN0M0 |
| 27 | Male | 60 | BCa | TaN0M0 |
| 28 | Male | 74 | BCa | T2N0M0 |
| 29 | Female | 82 | BCa | T3N2M0 |
| 30 | Female | 71 | BCa | T1N0M0 |
| 31 | Female | 65 | BCa | T3N0M0 |
| 32 | Male | 82 | BCa | T1N0M0 |
| 33 | Male | 60 | BCa | T1N0M0 |
| 34 | Male | 67 | BCa | TaN0M0 |
| 35 | Female | 77 | BCa | TaN0M0 |
| 36 | Male | 70 | BCa | T3N2M0 |
| 37 | Male | 70 | BCa | T2N0M0 |
| 38 | Male | 55 | BCa | TaN0M0 |
| 39 | Female | 83 | BCa | T2N0M0 |
| 40 | Male | 69 | BCa | T2N0M0 |
| 41 | Male | 68 | BCa | T1N0M0 |
| 42 | Male | 66 | BCa | TaN0M0 |
| 43 | Male | 66 | BCa | T3N1M0 |
| 44 | Female | 70 | BCa | T1N0M0 |
| 45 | Male | 70 | BCa | T3N1M0 |
| 44 | Female | 64 | BCa | T1N0M0 |
| 45 | Male | 81 | BCa | T1N0M0 |
| 46 | Male | 64 | BCa | T1N0M0 |
| 47 | Female | 65 | BCa | TaN0M0 |
| 48 | Male | 70 | BCa | T2N0M0 |
| 49 | Male | 61 | BCa | T3N2M0 |
| 50 | Male | 81 | BCa | T2N0M0 |
| 51 | Male | 69 | BCa | T2N1M0 |
| 52 | Male | 77 | BCa | TaN0M0 |
| 53 | Male | 65 | BCa | T2N0M0 |
| 54 | Female | 77 | BCa | T3N2M0 |
| 55 | Male | 60 | BCa | T2N1M0 |
| 56 | Male | 60 | PCa | T4N1M1 |
| 57 | Male | 54 | PCa | T2N0M0 |
| 58 | Male | 53 | PCa | T4N1M0 |
| 59 | Male | 71 | PCa | T2N0M0 |
| 60 | Male | 67 | PCa | T3N0M0 |
| 61 | Male | 54 | PCa | T2N0M0 |
| 62 | Male | 65 | PCa | T3N0M0 |
| 63 | Male | 83 | PCa | T4N0M1 |
| 64 | Male | 80 | PCa | T4N0M1 |
| 65 | Male | 70 | PCa | T3N0M0 |
| 66 | Male | 70 | PCa | T2N0M0 |
| 67 | Male | 36 | RCa | T1N0M0 |
| 68 | Male | 60 | RCa | T3N0M0 |
| 69 | Male | 70 | RCa | T3N0M0 |
| 70 | Female | 70 | RCa | T3N0M0 |
| 71 | Male | 53 | RCa | T1N0M0 |
| 72 | Female | 75 | RCa | T1N0M0 |
| 73 | Male | 44 | RCa | T3N0M0 |
| 74 | Male | 68 | RCa | T1N0M0 |
| 75 | Male | 36 | RCa | T1N0M0 |
| 76 | Male | 69 | RCa | T1N0M0 |
| 77 | Male | 67 | RCa | T2N0M0 |
| 78 | Male | 61 | RCa | T1N0M0 |
| 79 | Male | 68 | RCa | T1N0M0 |
| 80 | Male | 63 | RCa | T1N0M0 |
| 81 | Male | 67 | RCa | T3N0M0 |
| 82 | Female | 58 | RCa | T3N1M0 |
| 83 | Female | 45 | RCa | T1N0M0 |
| 84 | Female | 53 | Healthy | |
| 85 | Female | 58 | Healthy | |
| 86 | Male | 55 | Healthy | |
| 87 | Female | 26 | Healthy | |
| 88 | Male | 55 | Healthy | |
| 89 | Female | 26 | Healthy | |
| 90 | Male | 22 | Healthy | |
| 91 | Male | 23 | Healthy | |
| 92 | Male | 25 | Healthy | |
| 93 | Male | 29 | Healthy | |
| 94 | Female | 28 | Healthy | |
| 95 | Male | 31 | Healthy | |
| 96 | Female | 29 | Healthy | |
| 97 | Male | 26 | Healthy | |
| 98 | Male | 27 | Healthy | |
| 99 | Male | 27 | Healthy | |
| 100 | Male | 32 | Healthy | |
| 101 | Male | 26 | Healthy | |
| 102 | Male | 25 | Healthy | |
| 103 | Female | 25 | Healthy | |
| 104 | Male | 25 | Healthy | |

Note: BCa, bladder cancer patient; PCa, prostate cancer patient; RCa, renal cancer patient.

Table S2. Diagnostic performance of five diagnostic logics

|  |  | HV | | ES | | TS | |
| --- | --- | --- | --- | --- | --- | --- | --- |
|  | Total Accuracy | Sensitivity | Precision | Sensitivity | Precision | Sensitivity | Precision |
| Method 1 | 0.7632 | 0.9091 | 1 | 1 | 0.6538 | 0.2 | 1 |
| Method 2 | 0.7895 | 1 | 0.9167 | 0.5294 | 1 | 1 | 0.5882 |
| Method 3 | 0.9211 | 0.8182 | 1 | 0.9412 | 0.8889 | 1 | 0.9091 |
| Method 4 | 0.8947 | 0.9091 | 1 | 1 | 0.8095 | 0.7 | 1 |
| This method | 0.9737 | 1 | 1 | 0.9412 | 1 | 1 | 0.9091 |

Note: HV, healthy volunteer; ES, early-stage patients; TS, terminal-stage patients.

Table S3. Summary of the information of 19 signatures

| No. | Raman shift (cm^–1^) | band | Assignment | Contribution | Reference |
| --- | --- | --- | --- | --- | --- |
| n1 | 485-487 | Glycans | Glycogen | 0.44 | [6] |
| ta1 | 584-585 |  | O‒P‒O single asymmetric bending mode | 0.53 | [7] |
| n2 | 587-589 | Glycerol |  | 0.55 | [8] |
| n3 | 607-608 | Lipids | Cholesterol | 0.45 | [9] |
| n4 | 611-613 | Lipids | Cholesterol /Cholesterol ester | 0.90 | [10] |
| ta2 | 619-620 | Protein | Phenylalanine C‒C twist aromatic ring | 0.50 | [11] |
| n5 | 645-646 | Protein | tyrosine | 0.50 | [12] |
| n6 | 664-666 | Nucleic acid | Ring breathing modes in the DNA bases | 0.44 | [13] |
| n7 | 669-671 | Nucleic acid | thymine | 0.57 | [14] |
| ta3 | 684 | Nucleic acid | Intense ring breathing modes of guanine | 0.53 | [15] |
| n8 | 701-703 | Lipids | Cholesterol | 0.43 | [9] |
| n9 | 722 | Nucleic acid | DNA | 0.43 | [14] |
| ta4 | 733-740 | Lipids | Phosphatidylserine | 0.52 | [8] |
| ta5 | 746 | Nucleic acid | ‒O‒C=O bending vibration in thymine | 0.49 | [16] |
| n10 | 855-859 | Protein | Collagen | 0.69 | [17] |
| ta6 | 1016 | Glycans | Carbohydrates | 0.49 | [18] |
| ta7 | 1065-1076 | Protein | Phosphate vibrations | 0.85 | [19] |
| ta8 | 1118-1123 | Protein | C‒C stretching mode of lipids and protein, C‒N stretch | 0.79 | [20] |
| ta9 | 1420-1421 | Protein | β-sheet | 0.54 | [21] |

Note: Columns highlighted with yellow refer to nEV feature information, while those highlighted with red shows taEV feature information.

**References**

[1] J. Turkevich, P. C. Stevenson, J. Hillier, *Discuss. Faraday Soc.* **1951**, *11*, 55.

[2] M. Weiler, S. B. Quint, S. Klenk, C. Pacholski, *Chem Commun* **2014**, *50*, 15419–15422.

[3] S. Dong, Y. Wang, Z. Liu, W. Zhang, K. Yi, X. Zhang, X. Zhang, C. Jiang, S. Yang, F. Wang, X. Xiao, *ACS Appl. Mater. Interfaces* **2020**, *12*, 5136–5146.

[4] A. Minopoli, B. Della Ventura, B. Lenyk, F. Gentile, J. A. Tanner, A. Offenhäusser, D. Mayer, R. Velotta, *Nat. Commun.* **2020**, *11*, 6134.

[5] R. R. Selvaraju, M. Cogswell, A. Das, R. Vedantam, D. Parikh, D. Batra, *Int. J. Comput. Vis.* **2020**, *128*, 336–359.

[6] S. O. Konorov, H. G. Schulze, C. G. Atkins, J. M. Piret, S. A. Aparicio, R. F. B. Turner, M. W. Blades, *Anal. Chem.* **2011**, *83*, 6254–6258.

[7] R. Selvaraju, A. Raja, G. Thiruppathi, *Spectrochim. Acta. A. Mol. Biomol. Spectrosc.* **2012**, *99*, 205–210.

[8] C. Krafft, L. Neudert, T. Simat, R. Salzer, *Spectrochim. Acta. A. Mol. Biomol. Spectrosc.* **2005**, *61*, 1529–1535.

[9] C. Matthäus, S. Dochow, G. Bergner, A. Lattermann, B. F. M. Romeike, E. T. Marple, C. Krafft, B. Dietzek, B. R. Brehm, J. Popp, *Anal. Chem.* **2012**, *84*, 7845–7851.

[10] N. Bergner, A. Medyukhina, K. D. Geiger, M. Kirsch, G. Schackert, C. Krafft, J. Popp, *Anal. Bioanal. Chem.* **2013**, *405*, 8719–8728.

[11] A. Rygula, K. Majzner, K. M. Marzec, A. Kaczor, M. Pilarczyk, M. Baranska, *J. Raman Spectrosc.* **2013**, *44*, 1061–1076.

[12] S. Verrier, I. Notingher, J. M. Polak, L. L. Hench, *Biopolymers* **2004**, *74*, 157–162.

[13] D. Li, L. Xia, Q. Zhou, L. Wang, D. Chen, X. Gao, Y. Li, *Anal. Chem.* **2020**, *92*, 12769–12773.

[14] N. Rashid, H. Nawaz, K. W. C. Poon, F. Bonnier, S. Bakhiet, C. Martin, J. J. O’Leary, H. J. Byrne, F. M. Lyng, *Exp. Mol. Pathol.* **2014**, *97*, 554–564.

[15] J. Morla-Folch, R. A. Alvarez-Puebla, L. Guerrini, *J. Phys. Chem. Lett.* **2016**, *7*, 3037–3041.

[16] V. Karunakaran, V. N. Saritha, M. M. Joseph, J. B. Nair, G. Saranya, K. G. Raghu, K. Sujathan, K. S. Kumar, K. K. Maiti, *Nanomedicine Nanotechnol. Biol. Med.* **2020**, *29*, 102276.

[17] L. Becker, C.-E. Lu, I. A. Montes-Mojarro, S. L. Layland, S. Khalil, A. Nsair, G. P. Duffy, F. Fend, J. Marzi, K. Schenke-Layland, *Acta Biomater.* **2023**, *162*, 278–291.

[18] F. M. Eltigani, X. Zhang, M. Liu, J. Peng, X. Su, *Opt. Laser Technol.* **2024**, *178*, 111208.

[19] S. Siddhanta, D. Karthigeyan, P. P. Kundu, T. K. Kundu, C. Narayana, *RSC Adv.* **2013**, *3*, 4221.

[20] X. Cheng, H. Liang, Q. Li, J. Wang, J. Liu, Y. Zhang, Y. Ru, Y. Zhou, *Spectrochim. Acta. A. Mol. Biomol. Spectrosc.* **2022**, *281*, 121558.

[21] T.-H. Liu, M. Okuno, *J. Phys. Chem. B* **2023**, *127*, 6675–6683.
